# Comparison of Artificial Intelligence-Driven Echocardiographic Assessment of Diastolic Function With the ASE Guidelines

**DOI:** 10.1016/j.jacadv.2026.103115

**Published:** 2026-09-23

**Authors:** Márton Tokodi, Nobuyuki Kagiyama, Ambarish Pandey, Yutaka Nakamura, Yuka Akama, Sachiko Takamatsu, Misako Toki, Takeshi Kitai, Taiji Okada, Carolyn S.P. Lam, Naveena Yanamala, Partho P. Sengupta

**Affiliations:** a Heart and Vascular Center, Semmelweis University, Budapest, Hungary; b Department of Experimental Cardiology and Surgical Techniques, Semmelweis University, Budapest, Hungary; c Department of Cardiovascular Biology and Medicine, Juntendo University Graduate School of Medicine, Tokyo, Japan; d AI Incubation Farm, Juntendo University Faculty of Medicine, Tokyo, Japan; e Division of Cardiology, Department of Internal Medicine, University of Texas Southwestern Medical Center, Dallas, Texas, USA; f Department of Nursing, The Sakakibara Heart Institute of Okayama, Okayama, Japan; g Department of Clinical Laboratory, The Sakakibara Heart Institute of Okayama, Okayama, Japan; h Department of Cardiovascular Medicine, Kobe City Medical Center General Hospital, Kobe, Japan; i Department of Heart Failure, National Cerebral and Cardiovascular Center, Suita, Japan; j Division of Cardiology, Shimane University Faculty of Medicine, Izumo, Japan; k National Heart Centre Singapore and Duke-National University of Singapore, Singapore; l Division of Cardiovascular Diseases and Hypertension, Department of Medicine, Rutgers Robert Wood Johnson Medical School, New Brunswick, New Jersey, USA; m Department of Cardiology, Mayo Clinic Health System, Eau Claire, Wisconsin, USA

**Keywords:** echocardiography, diastolic dysfunction, deep learning, left ventricular filling pressure, prognosis

## Abstract

**Background:**

Contemporary guideline-based assessment of left ventricular diastolic function and filling pressure requires multiple echocardiographic parameters that may not be routinely acquired in real-world clinical practice.

**Objectives:**

The aim of the study was to compare the prognostic and diagnostic performance of a previously validated deep learning (DL) model for left ventricular diastolic function assessment with those of the 2016 ASE (American Society of Echocardiography)/EACVI (European Association of Cardiovascular Imaging) and 2025 ASE guidelines.

**Methods:**

The DL model and the guidelines were compared in the ARIC (Atherosclerosis Risk In Communities) cohort (n = 5,450) for prognostication and in 2 hemodynamic validation cohorts from the United States (n = 83) and Japan (n = 130) for diagnosing elevated left ventricular filling pressure. In all 3 cohorts, ≥1 guideline-recommended parameters were unavailable in a substantial proportion of patients.

**Results:**

In the ARIC cohort, the DL model achieved a higher C-index than both the 2016 ASE/EACVI and 2025 ASE guidelines (0.676 vs 0.638 and 0.602, respectively; both *P* < 0.001). In the United States cohort, the DL model achieved a higher area under the receiver operating characteristic curve (AUC) than the 2025 ASE guidelines (0.879 vs 0.822; *P* = 0.041) and a similar AUC to the 2016 ASE/EACVI guidelines (0.879 vs 0.812; *P* = 0.138). In the Japanese cohort, the DL model achieved a higher AUC than both guideline versions (0.816 vs 0.694 and 0.634, respectively; both *P* < 0.05).

**Conclusions:**

In settings where not all guideline-recommended parameters are available, the DL model may improve prognostic and diagnostic performance compared with both guideline versions. Nevertheless, these exploratory findings require further validation to confirm that the observed advantages persist when all guideline-recommended parameters are systematically acquired.

Left ventricular (LV) diastolic dysfunction (DD) is a common and prognostically significant abnormality in patients with cardiovascular diseases, contributing to symptoms of heart failure (HF), reduced exercise tolerance, and adverse outcomes, even in the presence of preserved left ventricular ejection fraction (LVEF).^1^^,^^2^ Given the central role of elevated left ventricular filling pressure (LVFP) in the pathophysiology and clinical consequences of DD, accurate assessment of diastolic function is essential for contemporary HF care and risk stratification.

To guide this assessment, the ASE (American Society of Echocardiography) and the EACVI (European Association of Cardiovascular Imaging) developed guidelines that define stepwise decision algorithms based on echocardiographic parameters.^3^, ^4^, ^5^, ^6^ The 2016 recommendations provided a pragmatic framework but showed modest correlation with invasively measured LVFP and a high rate of indeterminate results.^7^, ^8^, ^9^, ^10^, ^11^ The updated 2025 ASE guidelines introduced additional Doppler and strain parameters, along with refined decision pathways, to reduce indeterminate classifications and improve diagnostic accuracy.^5^^,^^12^ However, implementation depends on complex measures that are not consistently available in routine clinical practice.^12^^,^^13^

Rule-based guideline algorithms rely on fixed cutoffs and may not capture the multivariable, nonlinear, and age-dependent nature of diastolic physiology.^14^ In contrast, we have previously shown that machine learning and deep learning (DL) approaches can integrate multiple echocardiographic features, model age-related variation, and improve the prediction of outcomes and pathophysiologic states across diverse cohorts.^15^, ^16^, ^17^, ^18^ More importantly, because these approaches often rely only on routinely acquired echocardiographic parameters, they can also be applied in settings where comprehensive acquisition of all guideline-recommended parameters for diastolic function assessment is not feasible.

Accordingly, we compared a validated DL model with the 2016 ASE/EACVI and 2025 ASE guidelines in participants of the ARIC (Atherosclerosis Risk In Communities) cohort study for prognostication and in 2 geographically distinct cohorts from the United States and Japan for the detection of elevated LVFP. We hypothesized that the DL model, which was designed to extract latent physiologic traits from routinely acquired echocardiographic variables, would match or perhaps even surpass the diagnostic and prognostic performance of contemporary guideline-based algorithms in settings where ≥1 guideline-recommended parameters are unavailable.

## Methods

### Validation cohort for predicting clinical outcomes: the ARIC cohort

**Figure 1:**
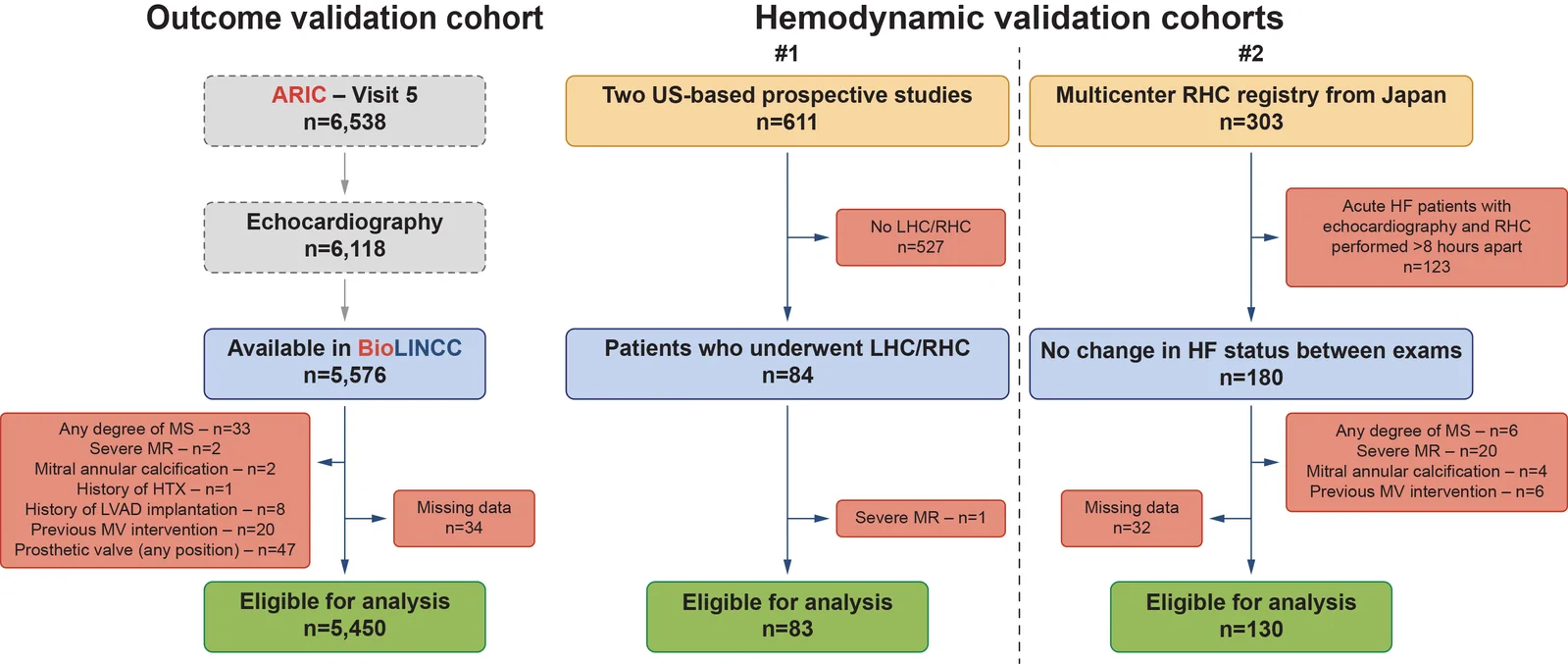
Patient Selection Flowcharts of the 3 Cohorts Included in the Study ARIC = Atherosclerosis Risk In Communities; BioLINCC = Biologic Specimen and Data Repository Information Coordinating Center; HF = heart failure; HTX = heart transplantation; LHC = left heart catheterization; LVAD = left ventricular assist device; MR = mitral regurgitation; MS = mitral stenosis; MV = mitral valve; RHC = right heart catheterization.

We validated the prognostic value of the guidelines and the DL model using data collected in the ARIC cohort study.^19^ We analyzed the data of those participants who underwent a transthoracic echocardiographic examination at visit 5 between 2011 and 2013 and whose data were available in the Biologic Specimen and Data Repository Information Coordinating Center database (Figure 1). From the 5,576 participants who fulfilled these criteria, we excluded those with any degree of mitral stenosis (n = 33), severe mitral regurgitation (n = 2), documented mitral annular calcification (n = 2), a history of heart transplantation (n = 1), a history of LV assist device implantation (n = 8), previous mitral valve interventions (n = 20), or a prosthetic valve at any position (n = 47). In addition, we also excluded participants with >3 missing values in key echocardiographic variables (ie, the input features of the DL model; n = 31) or no follow-up data (n = 3). The echocardiographic protocol of visit 5 has been published previously^20^ and is described briefly in the Supplemental Methods. H_2_FPEF score was calculated for participants with preserved LVEF (≥50%) according to the original published algorithm,^21^ as detailed in the Supplemental Methods. The primary outcome of interest was the composite of HF hospitalization or all-cause death, but the main results are also reported for HF hospitalization and all-cause death separately. The time to event was measured from the date of the echocardiographic examination at visit 5.

### Validation cohorts for detecting elevated LVFP

We validated the guidelines and the DL model against invasively measured LVFP in 2 distinct cohorts of patients with paired echocardiographic and invasive hemodynamic studies (Figure 1). The first cohort comprised patients with varying degrees of systolic dysfunction and DD who underwent transthoracic echocardiography and invasive hemodynamic assessment either with left heart catheterization (LHC) or right heart catheterization (RHC) when enrolled in 2 prospective studies at West Virginia University (Morgantown, West Virginia).^22^^,^^23^ From the 84 participants who fulfilled these criteria, we excluded those with severe mitral regurgitation (n = 1). None of the patients met the other exclusion criteria listed for the ARIC cohort, as they were prospectively enrolled in clinical studies with inclusion and exclusion criteria previously defined to permit echocardiographic assessment of diastolic function.^22^^,^^23^ The protocols of echocardiographic examinations and invasive hemodynamic evaluations have been described previously.^22^^,^^23^

The second hemodynamic validation cohort was derived from a Japanese retrospective multicenter registry that included 303 adults (≥20 years) who underwent both transthoracic echocardiography and RHC within 24 hours at Juntendo University (Tokyo, Japan), the Sakakibara Heart Institute of Okayama (Okayama, Japan), or the Kobe City Medical Center General Hospital (Kobe, Japan). To minimize errors resulting from changes in HF status between the 2 examinations, patients hospitalized for acute HF in whom echocardiography and RHC were performed more than 8 hours apart were excluded from the present study (n = 123). Patients with any degree of mitral stenosis (n = 6), severe mitral regurgitation (n = 20), mitral annular calcification (n = 4), previous mitral valve interventions (n = 6), >3 missing values in key echocardiographic variables (ie, the input features of the DL model; n = 28), or missing pulmonary capillary wedge pressure (n = 4) were also excluded. Echocardiographic examinations were performed in accordance with the current ASE/EACVI guidelines.^24^ RHC was performed via the jugular or femoral vein using a 5- to 8-Fr balloon-tipped, fluid-filled Swan-Ganz catheter (Edwards Lifesciences). Intracardiac pressures, including pulmonary capillary wedge pressure, were assessed under fluoroscopic guidance after calibration with 0 being positioned at the mid-thoracic line.

In the hemodynamic validation cohorts, elevated LVFP was defined as an RHC-derived pulmonary capillary wedge pressure or an LHC-derived LV preatrial contraction pressure >15 mm Hg.

### Guideline-based echocardiographic assessment of diastolic function

Diastolic function was assessed in all 3 cohorts according to the 2016 ASE/EACVI guidelines^4^ and the 2025 ASE guidelines.^5^

Using the 2016 ASE/EACVI guidelines, patients were classified into 5 categories: 1) normal diastolic function; 2) indeterminate diastolic function; 3) DD with normal left atrial pressure (LAP) (ie, grade 1 DD); 4) DD with indeterminate LAP; and 5) DD with elevated LAP (ie, grade 2 or 3 DD).

According to the 2025 ASE guidelines, patients were assigned to 1 of 4 categories: 1) normal diastolic function; 2) DD with normal LAP; 3) DD with indeterminate LAP; and 4) DD with elevated LAP. Although the 2025 ASE guidelines were developed to potentially reduce the number of cases with indeterminate assessment, patients were still classified as having DD with indeterminate LAP: 1) if they were in sinus rhythm, only early diastolic mitral annular velocity (eʹ) was reduced among the primary parameters, and the ratio of the early and late mitral inflow velocities (E/A) was unavailable; 2) if they were in sinus rhythm and additional parameters were required to assess LAP but none were available; 3) if they had noncardiac pulmonary hypertension and E/A was unavailable; 4) if they had noncardiac pulmonary hypertension, LAP could not be assessed solely based on E/A, left atrial reservoir strain (LARS) was unavailable, and lateral E/eʹ ranged from 8 to 13; 5) if they were in atrial fibrillation and <3 of the primary parameters were available; and 6) if they were in atrial fibrillation and exactly 2 of the primary criteria were met, but <2 of the secondary parameters were available.

The availability of the echocardiographic parameters used for diastolic function assessment is reported in Supplemental Table 1.

### DL-based assessment of diastolic function

We used our previously published and extensively validated DL model that assesses diastolic function based on 9 routinely measured echocardiographic parameters: LVEF, LV mass index, left atrial volume index, E, A, E/A, septal eʹ, septal E/eʹ, and tricuspid regurgitation peak velocity.^15^^,^^25^ The output of the DL model is a single numeric value for each subject, denoting the probability of DD. One of the algorithm’s significant advantages is its ability to provide accurate diagnostic and prognostic predictions even when input echocardiographic variables contain missing values. Further details on the DL model and its external validation are provided in the Supplemental Methods and have been published previously.^15^ The model is publicly available online.^26^

### Statistical analysis

Continuous variables are expressed as median (Q1-Q3), whereas categorical variables are reported as frequencies and percentages. The normality of continuous variables was checked using the Shapiro-Wilk test. The characteristics of patient subgroups were compared using unpaired Student’s *t*-test or Mann-Whitney *U* test for continuous variables and chi-square or Fisher exact test for categorical variables, as appropriate. The event-free survival of the subgroups was visualized using Kaplan-Meier curves, and pairwise log-rank tests with the Benjamini-Hochberg adjustment were applied for comparison. For the composite endpoint and all-cause death, Cox proportional hazards models were used to compute HRs with 95% CIs. For HF hospitalization, subdistribution HRs with 95% CIs were estimated using Fine-Gray competing risks regression, with all-cause death treated as the competing event. The proportional hazards assumption was verified by the absence of systematic patterns in Schoenfeld residuals for each covariate. To compare the prognostic value of the DL model and the guidelines, Harrell’s C-indices of Cox regression models in the same patients were compared using the *compareC* R package.^27^ Bootstrap resampling (with 10,000 stratified resamples) was applied to calculate *P* values when comparing C-indices derived from Cox regression models in different patient subsets or from Fine-Gray competing risk models. Time-dependent receiver operating characteristic (ROC) analysis was also performed to support and strengthen the results of the C-index comparisons. The performance of the guidelines and the DL model in detecting elevated LVFP was assessed using the area under the receiver operating characteristic curve (AUC). Because the guideline-based classifications were nominal, univariable logistic regression models were fitted in which the classifications were used as predictors of the binary reference standard (ie, elevated LVFP). Through this approach, the nominal classifications were converted into continuous probability estimates, enabling the calculation of AUCs and allowing for direct comparison with the DL model using DeLong tests. A value of *P* < 0.05 was considered statistically significant. All statistical analyses were performed in R (version 4.5.2; R Foundation for Statistical Computing).

### Ethical approval

All participants of the ARIC cohort study provided written informed consent, and the institutional review boards associated with each field center approved the study protocol. The 2 United States-based prospective studies used for hemodynamic validation were approved by the corresponding institutional review boards, and written informed consent was obtained from all patients. Data collection and analysis for the multicenter Japanese registry were approved by the institutional review boards of each center. Given the retrospective observational design, written informed consent was waived, and an opt-out consent procedure was implemented. The protocol for the current analysis adheres to the principles outlined in the Declaration of Helsinki and was approved by the Institutional Review Board of Rutgers Biomedical and Health Sciences (study identification number: Pro2021001505). Methods and results are reported in compliance with the updated PRIME 2.0 (Proposed Requirements for Cardiovascular Imaging-Related Multimodal-AI Evaluation) checklist (Supplemental Table 2).^28^

## Results

### Prognostic value in the entire ARIC cohort

**Figure 2:**
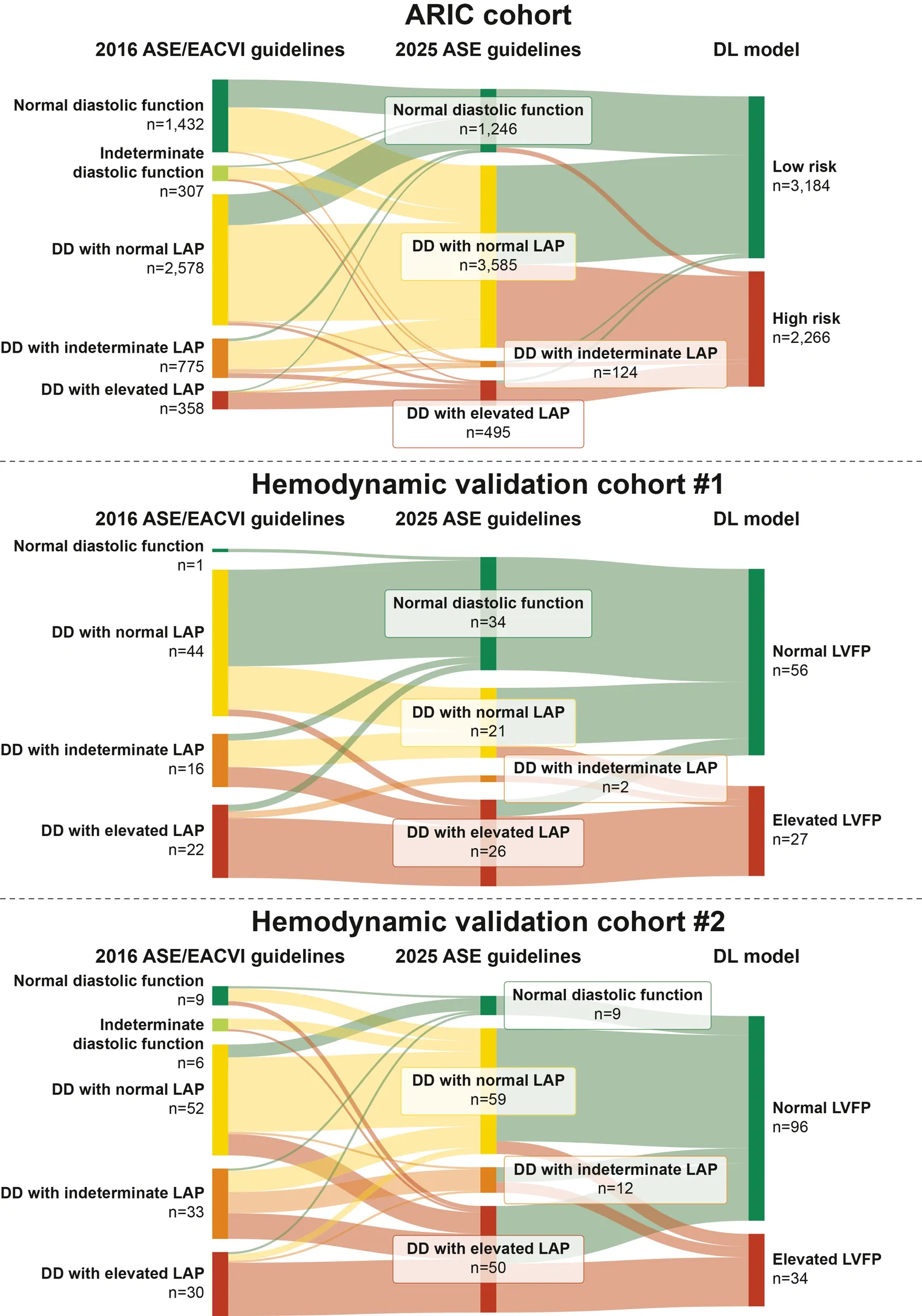
Sankey Diagrams Depicting the Reclassification of Patients Between the Guidelines and the DL Model Given that the DL model was primarily trained for prognostication, the default probability threshold of 0.5 was used to classify patients as high risk or low risk in the ARIC cohort but not in the hemodynamic validation cohorts. Instead, in the first hemodynamic validation cohort, a threshold specifically optimized for identifying elevated LVFP was derived using Youden’s J statistic, yielding a cutoff of 0.99957. To preserve this operating point while avoiding cohort-specific optimization in the second hemodynamic validation cohort, percentile-based threshold transport was applied. Accordingly, the top 32.5% of DL-predicted probabilities were classified as having elevated LVFP, corresponding to a cohort-specific numeric cutoff of 0.98612. ASE = American Society of Echocardiography; DD = diastolic dysfunction; DL = deep learning; EACVI = European Association of Cardiovascular Imaging; LAP = left atrial pressure; LVFP = left ventricular filling pressure; other abbreviations as in Figure 1.

Of the 5,450 ARIC study participants eligible for analysis, 1,320 (24%) reached the composite endpoint of HF hospitalization or all-cause death over 6.5 years (Q1-Q3: 6.1-7.0 years). As shown in Figure 2, reclassifications were observed between the 3 approaches, with significantly fewer patients classified as indeterminate using the 2025 guidelines than the 2016 guidelines (124/5,450 [2%] vs 1,082/5,450 [20%]; *P* < 0.001). The clinical and echocardiographic characteristics of the ARIC cohort are reported in Table 1 and Supplemental Table 3.

**Table 1:** Clinical and Echocardiographic Characteristics of the 3 Cohorts

	ARIC Cohort (Visit 5) (n = 5,450)	Hemodynamic Validation Cohort #1 (n = 83)	Hemodynamic Validation Cohort #2 (n = 130)
Demographics, vitals, risk factors			
Age, y	75 (71-79)	61 (54-66)	66 (51-75)
Male	2,326 (43)	56 (67)	70 (54)
Black race	1,049 (19)	3 (4)	0 (0)
BMI, kg/m^2^	27.9 (24.9-31.5)	30.8 (27.6-34.7)	22.0 (19.4-24.7)
BSA, m^2^	1.86 (1.70-2.01)	2.11 (1.94-2.28)	1.58 (1.43-1.73)
SBP, mm Hg	129 (118-141)	133 (117-147)	112 (100-126)
DBP, mm Hg	66 (59-74)	77 (69-89)	65 (57-76)
HR, 1/min	64 (57-71)	73 (65-85)	70 (62-80)
Hypertension	4,391 (81)	82 (99)	49 (38)
Diabetes	2,004 (37)	42 (51)	30 (23)
Atrial fibrillation	166 (3)	6 (7)	21 (16)
Chronic kidney disease	1,500 (28)	14 (17)	—
History of HF	908 (17)	30 (36)	—
History of CHD	1,048 (19)	67 (81)	29 (22)
Laboratory results			
BNP, pg/mL	—	1,068 (425-2,170)	168 (45-318)
NT-proBNP, pg/mL	134 (69-264)	—	326 (192-1,282)
Echocardiographic parameters			
IVSd, mm	10 (9-11)	11 (11-14)	10 (9-11)
LVPWd, mm	9 (8-10)	10 (9-12)	10 (9-11)
LVIDd, mm	44 (41-47)	46 (42-50)	47 (41-56)
LVIDs, mm	26 (23-29)	33 (29-35)	31 (26-45)
LVMi, g/m^2^	76.4 (66.1-89.1)	99.0 (80.8-118.3)	102.5 (81.5-141.8)
LVEDVi, mL/m^2^	42.4 (36.4-49.7)	57.3 (47.7-76.0)	74.2 (56.2-97.5)
LVESVi, mL/m^2^	14.4 (11.6-17.9)	26.9 (19.6-40.3)	32.3 (16.3-71.7)
LVEF, %	65.8 (61.9-69.2)	54.0 (38.0-59.5)	61.9 (37.3-69.9)
LAVi, mL/m^2^	24.7 (20.3-30.2)	27.9 (20.7-38.7)	35.5 (25.1-48.6)
LARS, %	—	21.4 (11.0-30.5)	17.2 (10.7-27.8)
Moderate or greater AS	40 (1)	3 (4)	8 (6)
Moderate or greater AR	22 (0)	1 (1)	10 (8)
Moderate MR	98 (2)	11 (13)	21 (16)
E, cm/s	65 (54-78)	81 (63-95)	70 (51-90)
E-DT, ms	200 (173-230)	202 (192-232)	192 (150-238)
A, cm/s	79 (67-91)	70 (56-85)	70 (49-84)
E/A	0.8 (0.7-1.0)	1.0 (0.8-1.5)	0.9 (0.6-1.4)
IVRT, ms	—	77 (70-90)	89 (73-114)
eʹ (septal), cm/s	5.5 (4.7-6.5)	6.0 (4.7-7.3)	5.5 (3.7-7.3)
E/eʹ (septal)	11.6 (9.5-14.4)	12.7 (9.5-20.0)	13.1 (9.7-16.8)
eʹ (lateral), cm/s	6.8 (5.6-8.2)	8.0 (6.0-9.9)	8.4 (6.7-11.4)
E/eʹ (lateral)	9.5 (7.6-12.0)	9.1 (6.9-14.6)	8.3 (5.9-10.8)
eʹ (average), cm/s	6.2 (5.3-7.3)	7.5 (5.5-8.4)	7.3 (5.6-9.2)
E/eʹ (average)	10.6 (8.7-13.1)	10.1 (8.0-17.8)	9.2 (7.2-11.9)
TRV, m/s	2.36 (2.18-2.55)	2.54 (1.87-2.95)	2.69 (2.24-3.20)
PASP, mm Hg	—	24 (13-37)	37 (26-49)
2016 ASE/EACVI guidelines			
Normal diastolic function	1,432 (26)	1 (1)	9 (7)
Indeterminate diastolic function	307 (6)	0 (0)	6 (5)
DD with normal LAP	2,578 (47)	44 (53)	52 (40)
DD with indeterminate LAP	775 (14)	16 (19)	33 (25)
DD with elevated LAP	358 (7)	22 (27)	30 (23)
2025 ASE guidelines			
Normal diastolic function	1,246 (23)	34 (41)	9 (7)
DD with normal LAP	3,585 (66)	21 (25)	59 (45)
DD with indeterminate LAP	124 (2)	2 (2)	12 (9)
DD with elevated LAP	495 (9)	26 (31)	50 (38)
DL-predicted probability of DD	0.30 (0.04-0.86)	0.95 (0.21-1.00)	0.98 (0.60-0.99)
Invasive hemodynamic assessment			
LVFP, mm Hg	—	11.1 (7.7-17.5)	10.0 (7.0-14.8)
LVFP >15 mm Hg	—	25 (30)	30 (23)

Values are median (Q1-Q3) or n (%). All characteristics of the ARIC cohort shown in this table were collected during visit 5.

A = late mitral inflow velocity; AR = aortic regurgitation; ARIC = Atherosclerosis Risk In Communities; AS = aortic stenosis; ASE = American Society of Echocardiography; BMI = body mass index; BNP = brain natriuretic peptide; BSA = body surface area; CHD = coronary heart disease; DBP = diastolic blood pressure; DD = diastolic dysfunction; DL = deep learning; E = early mitral inflow velocity; EACVI = European Association of Cardiovascular Imaging; E-DT = E-wave deceleration time; eʹ = early diastolic mitral annular velocity; HF = heart failure; HR = heart rate; IVRT = isovolumic relaxation time; IVSd = thickness of the interventricular septum at end-diastole; LAP = left atrial pressure; LARS = left atrial reservoir strain; LAVi = left atrial volume index; LV = left ventricular; LVEDVi = left ventricular end-diastolic volume index; LVEF = left ventricular ejection fraction; LVESVi = left ventricular end-systolic volume index; LVFP = left ventricular filling pressure; LVIDd = left ventricular internal diameter at end-diastole; LVIDs = left ventricular internal diameter at end-systole; LVMi = left ventricular mass index; LVPWd = thickness of the left ventricular posterior wall at end-diastole; MR = mitral regurgitation; NT-proBNP = N-terminal pro-brain natriuretic peptide; PASP = pulmonary artery systolic pressure estimated by echocardiography; SBP = systolic blood pressure; TRV = tricuspid regurgitation peak velocity.

**Figure 3:**
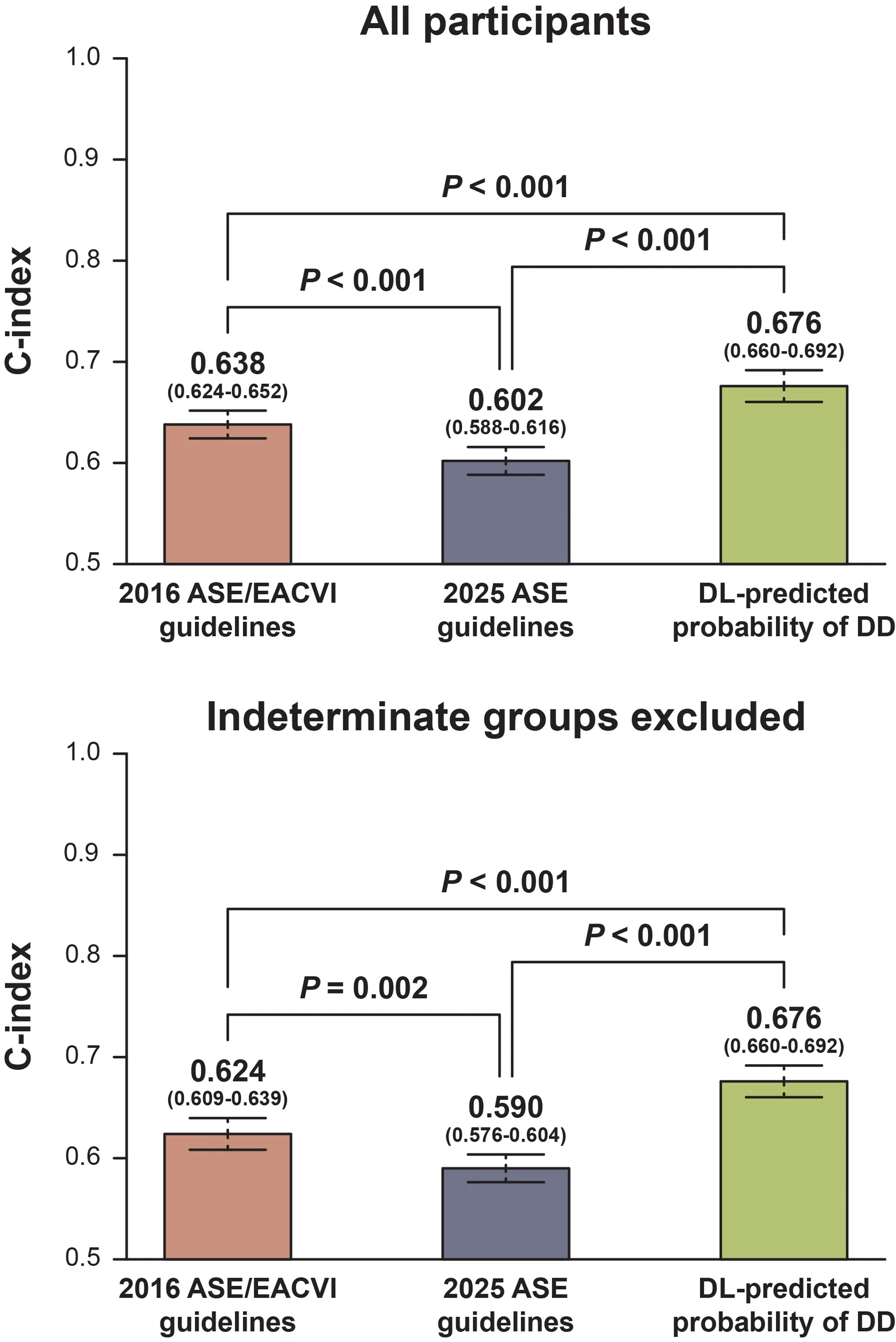
Prognostic Value of the Guidelines and the DL Model for Predicting the Composite Endpoint C-indices were calculated from univariable Cox regression models containing either the 2016 or 2025 guideline-based classification or the DL-predicted probability of DD. Models in the upper panel were evaluated in the entire ARIC cohort, whereas those in the lower panel were evaluated after excluding indeterminate groups. The 2 guidelines classified different patients as indeterminate; therefore, their performance was evaluated in overlapping but nonidentical subsets after excluding indeterminate cases: the 2016 ASE/EACVI guidelines were evaluated in 4,368/5,450 (80%) patients, whereas the 2025 ASE guidelines were evaluated in 5,326/5,450 (98%) patients. *P* values for C-index comparison were calculated using the *CompareC* R package^27^ in the upper panel, whereas they were calculated using bootstrap resampling in the lower panel. C-indices are reported with 95% CIs. Abbreviations as in Figures 1 and 2.

The guideline-derived diastolic function categories, as well as the DL-predicted probabilities, were associated with the composite endpoint of HF hospitalization or all-cause death both in univariable and multivariable Cox regression analyses (Table 2, Supplemental Tables 4 and 5). Moreover, both guideline-based classifications and the DL-predicted probability exhibited incremental prognostic value over N-terminal pro-brain natriuretic peptide (Supplemental Table 6). Notably, the DL model demonstrated superior prognostic power over both guidelines in univariable Cox regression, with a C-index of 0.676 (95% CI: 0.660-0.692) vs 0.638 (95% CI: 0.624-0.652) for the 2016 guidelines and 0.602 (95% CI: 0.588-0.616) for the 2025 guidelines (*P* < 0.001 for both comparisons) (Figure 3), even after adjustment for cardiovascular risk factors in multivariable Cox regression, with a C-index of 0.739 (95% CI: 0.725-0.753) vs 0.732 (95% CI: 0.718-0.746) for the 2016 guidelines and 0.728 (95% CI: 0.714-0.742) for the 2025 guidelines (*P* = 0.030 and *P* < 0.001, respectively) (Supplemental Figure 1). The 2016 guidelines achieved a higher C-index than the 2025 guidelines in univariable analysis (*P* < 0.001) (Figure 3) but not after adjustment for risk factors in multivariable analysis (*P* = 0.058) (Supplemental Figure 1). These findings were further supported by the results of the time-dependent ROC analyses (Supplemental Figure 2, Supplemental Table 7). We also found consistent results across patient subgroups stratified by race, the presence of atrial fibrillation, history of HF or coronary heart disease, elevated N-terminal pro-brain natriuretic peptide levels, and the availability of tricuspid regurgitation peak velocity measurements (Supplemental Table 8). After excluding patients with indeterminate diastolic function or DD with indeterminate LAP, the DL model maintained superior prognostic discrimination compared to both guidelines, with a C-index of 0.676 (95% CI: 0.660-0.692) vs 0.624 (95% CI: 0.609-0.639) for the 2016 guidelines and 0.590 (95% CI: 0.576-0.604) for the 2025 guidelines (*P* < 0.001 for both comparisons), and the 2016 guidelines also outperformed the 2025 guidelines (*P* = 0.002) (Figure 3).

**Table 2:** Univariable and Multivariable Cox Regression Models Predicting the Composite Endpoint of HF Hospitalization or All-Cause Death in the ARIC Cohort

	Univariable Models		Multivariable Model #1 C-Index: 0.732 (95% CI: 0.718-0.746) AIC: 21,183		Multivariable Model #2 C-Index: 0.728 (95% CI: 0.714-0.742) AIC: 21,207		Multivariable Model #3 C-Index: 0.739 (95% CI: 0.725-0.753) AIC: 21,105	
	HR (95% CI)	*P* Value	HR (95% CI)	*P* Value	HR (95% CI)	*P* Value	HR (95% CI)	*P* Value
Age, y	1.106 (1.095-1.117)	<0.001	1.080 (1.069-1.092)	<0.001	1.081 (1.069-1.092)	<0.001	1.075 (1.064-1.087)	<0.001
Male	1.505 (1.351-1.676)	<0.001	1.364 (1.219-1.526)	<0.001	1.342 (1.200-1.500)	<0.001	1.286 (1.150-1.438)	<0.001
Hypertension	2.006 (1.696-2.372)	<0.001	1.125 (0.945-1.341)	0.186	1.161 (0.975-1.382)	0.094	1.078 (0.905-1.285)	0.400
Diabetes	1.710 (1.534-1.905)	<0.001	1.411 (1.263-1.577)	<0.001	1.417 (1.268-1.584)	<0.001	1.375 (1.230-1.537)	<0.001
Atrial fibrillation	2.769 (2.220-3.454)	<0.001	1.330 (1.032-1.715)	0.028	1.475 (1.152-1.889)	0.002	1.554 (1.240-1.949)	<0.001
Chronic kidney disease	1.902 (1.703-2.124)	<0.001	1.385 (1.235-1.552)	<0.001	1.404 (1.252-1.573)	<0.001	1.404 (1.253-1.574)	<0.001
History of HF	3.267 (2.912-3.666)	<0.001	2.019 (1.773-2.300)	<0.001	2.129 (1.872-2.422)	<0.001	2.037 (1.791-2.317)	<0.001
History of CHD	2.340 (2.085-2.627)	<0.001	1.209 (1.059-1.380)	0.005	1.284 (1.127-1.463)	<0.001	1.206 (1.058-1.376)	0.005
History of noncardiac PH	3.761 (2.970-4.763)	<0.001	1.778 (1.393-2.270)	<0.001	1.427 (1.093-1.863)	0.009	1.690 (1.324-2.157)	<0.001
History of stroke	2.203 (1.780-2.726)	<0.001	1.142 (0.918-1.420)	0.234	1.106 (0.889-1.377)	0.366	1.062 (0.853-1.321)	0.591
2016 ASE/EACVI guidelines								
Indeterminate diastolic function	1.549 (1.146-2.094)	0.004	1.340 (0.989-1.815)	0.059				
DD with normal LAP	2.212 (1.868-2.620)	<0.001	1.394 (1.165-1.669)	<0.001				
DD with indeterminate LAP	3.905 (3.235-4.714)	<0.001	1.987 (1.609-2.455)	<0.001				
DD with elevated LAP	5.624 (4.558-6.938)	<0.001	2.712 (2.164-3.399)	<0.001				
2025 ASE guidelines								
DD with normal LAP	1.593 (1.361-1.865)	<0.001			1.243 (1.059-1.459)	0.008		
DD with indeterminate LAP	5.067 (3.847-6.674)	<0.001			1.680 (1.207-2.338)	0.002		
DD with elevated LAP	4.141 (3.423-5.010)	<0.001			2.202 (1.801-2.693)	<0.001		
DL-predicted probability of DD	4.632 (4.000-5.364)	<0.001					2.673 (2.293-3.117)	<0.001

The AIC values of the univariable models containing the 2016 ASE/EACVI guideline classifications, the 2025 ASE guideline classifications, and the DL-predicted probability of DD were 21,786, 21,860, and 21,683, respectively. Only clinically relevant variables without missing values were considered for the multivariable models.

AIC = Akaike information criterion; PH = pulmonary hypertension; other abbreviations as in Table 1.

**Figure 4:**
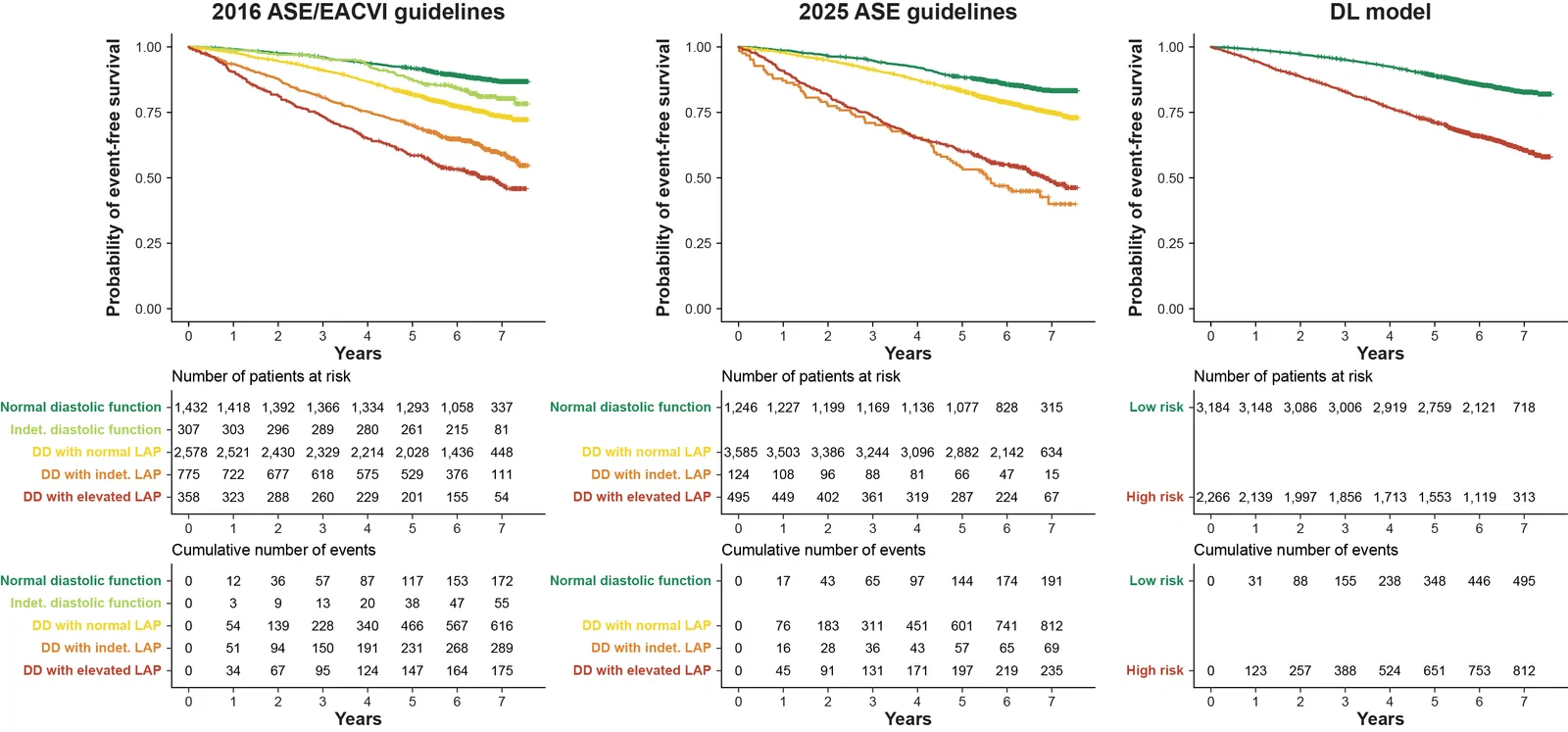
Kaplan-Meier Curves Showing the Event-Free Survival of Patient Subgroups in the ARIC Cohort The composite of HF hospitalization or all-cause death was used as the endpoint in this analysis. The *P* value from the log-rank test for the DL model was <0.001. *P* values from the pairwise log-rank tests of the guideline-based classifications are reported in Supplemental Tables 9 and 10. Abbreviations as in Figure2.

When we divided participants into 2 groups (ie, low-risk and high-risk groups) using the DL-predicted probability value of 0.5 as the cutoff and plotted their event-free survival, we observed that a higher proportion of high-risk compared to low-risk patients reached the composite endpoint during follow-up (Figure 4). We also observed a significant separation between the survival curves of patients categorized based on the 2016 and 2025 guidelines (Figure 4, Supplemental Tables 9 and 10).

The DL-predicted probabilities, as well as the guideline-based classifications, were also associated with HF hospitalization and all-cause death as separate endpoints in univariable and multivariable analyses (Supplemental Tables 11 and 12). Moreover, the DL model outperformed the 2 guidelines in predicting both of these endpoints (Supplemental Figure 3).

### Prognostic value in ARIC study participants with preserved LVEF

Of the 5,135 participants with available LVEF measurement at visit 5 and preserved LVEF, 1,170 (23%) reached the composite endpoint of HF hospitalization or all-cause death over 6.5 years (Q1-Q3: 6.1-7.0 years). In this subgroup of ARIC participants, the DL-predicted probability of DD demonstrated the highest prognostic discrimination (C-index: 0.660 [95% CI: 0.643-0.677]), significantly outperforming the 2016 (C-index: 0.628 [95% CI: 0.613-0.643]; *P* < 0.001) and 2025 guidelines (C-index: 0.590 [95% CI: 0.576-0.604]; *P* < 0.001) and the H_2_FPEF score (C-index: 0.607 [95% CI: 0.591-0.623]; *P* < 0.001) (Table 3). The 2016 guidelines also showed an improvement over the H_2_FPEF score (*P* = 0.014) (Table 3). In contrast, the 2025 ASE guidelines showed performance similar to that of the H_2_FPEF score (*P* = 0.070) (Table 3).

**Table 3:** Prognostic Value of the Guideline-Based Classifications and the DL Model Compared With the H_2_FPEF Score in ARIC Study Participants With Preserved LVEF

	C-Index	ΔC-Index	*P* Value
H_2_FPEF score	0.607 (0.591-0.623)		
2016 ASE/EACVI guidelines	0.628 (0.613-0.643)	+0.021 (0.005-0.039)	0.014
2025 ASE guidelines	0.590 (0.576-0.604)	−0.017 (−0.035-0.001)	0.070
DL-predicted probability of DD	0.660 (0.643-0.677)	+0.053 (0.034-0.072)	<0.001

Values are C-index (95% CI), unless otherwise indicated. Each row represents a univariable Cox regression model predicting the composite endpoint of HF hospitalization or all-cause death. The third column shows the difference in the C-index of the given model vs that of the model containing the H_2_FPEF score only, whereas the *P* value in the fourth column refers to the significance of this difference.

Abbreviations as in Table 1.

### Performance in detecting elevated LVFP

**Figure 5:**
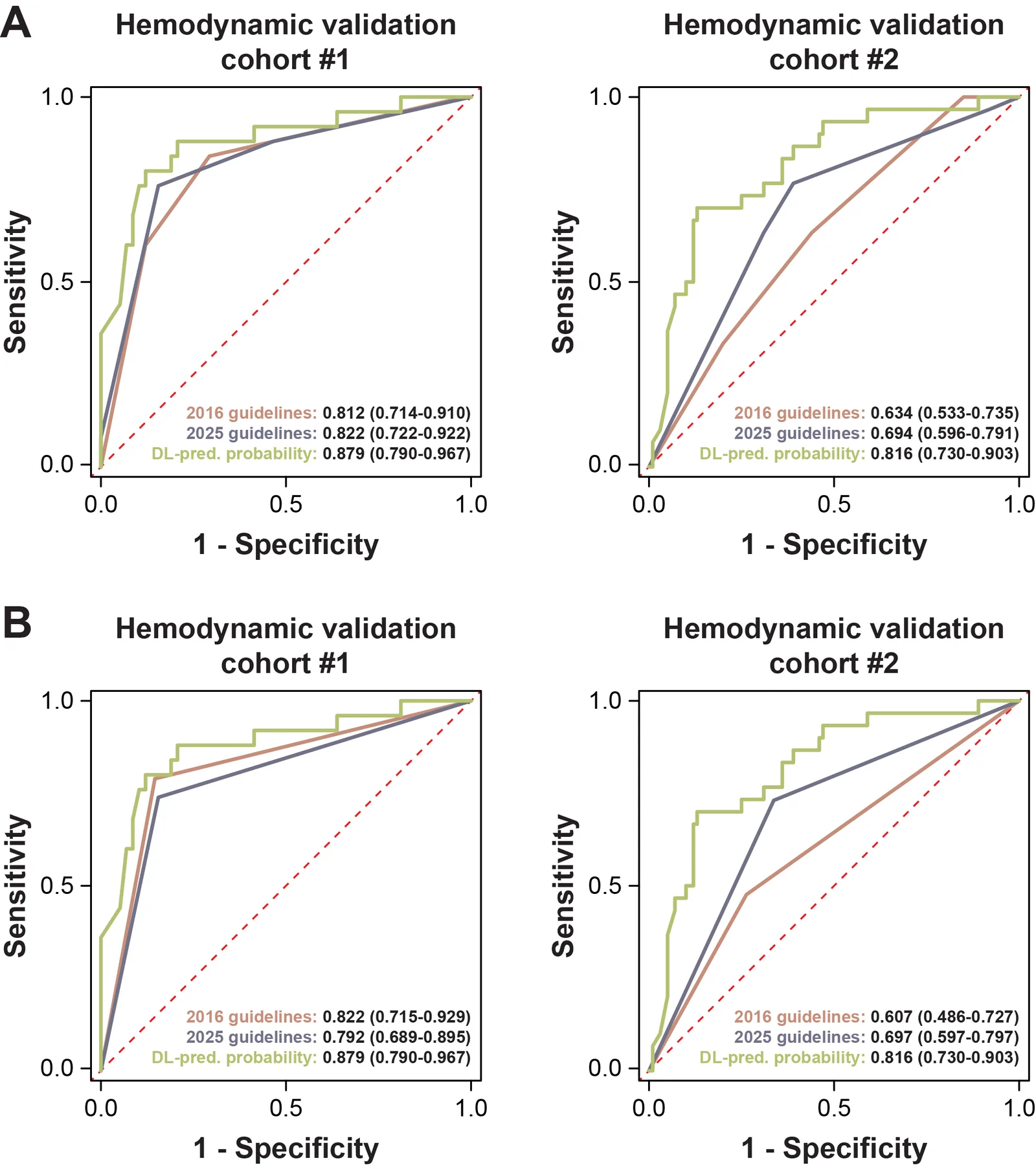
Receiver Operating Characteristic Curves Showing the Performance of the Guidelines and the DL Model in Detecting Elevated LVFP (A) ROC analysis was performed first for all patients in the given cohort. (B) Then, indeterminate groups were excluded, and ROC analyses were performed again in the remaining patients. Because the 2016 ASE/EACVI and 2025 ASE guidelines classified different sets of patients as indeterminate, their performance was assessed in overlapping but not identical patient subsets after excluding indeterminate cases: the 2016 guidelines were evaluated in 67/83 (81%) and 91/130 (70%) patients in the first and second hemodynamic validation cohorts, whereas the 2025 guidelines were evaluated in 81/83 (98%) and 118/130 (91%) patients, respectively. To enable the straightforward calculation of diagnostic performance metrics, both guideline-based classifications were dichotomized by merging the “normal diastolic function” and the “DD with normal LAP” groups (as both are supposed to have normal LAP). AUCs are reported with 95% CIs. Abbreviations as in Figure 2.

Of the 83 patients of the first hemodynamic validation cohort, 25 (30%) had elevated invasively measured LVFP. Invasive hemodynamic assessment was performed with LHC in 59 (71%) and RHC in 24 (29%) patients. Sixty-three (76%) patients underwent echocardiography and invasive hemodynamic assessment on the same day, and the remaining 20 (24%) underwent both tests within 72 hours (Table 1 and Supplemental Table 13). There were apparent reclassifications between the 3 methods, as shown by the Sankey diagram (Figure 2). Significantly fewer patients had DD with indeterminate LAP according to the 2025 guidelines than the 2016 guidelines (2/83 [2%] vs 16/83 [19%]; *P* < 0.001). In identifying patients with elevated LVFP, the DL model exhibited an AUC of 0.879 (95% CI: 0.790-0.967), which was higher than the AUC of the 2025 guidelines (0.822 [95% CI: 0.722-0.922]; *P* = 0.041) and numerically higher, although not statistically significant, than that of the 2016 guidelines (0.812 [95% CI: 0.714-0.910]; *P* = 0.138) (Figure 5A). There was no difference between the AUCs of the 2016 and 2025 guidelines (*P* = 0.815). The DL model outperformed both guidelines even after excluding patients with indeterminate diastolic function or DD with indeterminate LAP (Figure 5B, Table 4), patients with atrial fibrillation (Supplemental Figure 4), and those with both LARS and isovolumic relaxation time (IVRT) missing (Supplemental Figure 5).

**Table 4:** Performance of the Guidelines and the DL Model in Detecting Elevated LVFP in the First Hemodynamic Validation Cohort (Indeterminate Groups Excluded)

	2016 ASE/EACVI Guidelines	2025 ASE Guidelines	DL Model
DD with indeterminate LAP	16 (19)	2 (2)	—
AUC	0.822 (0.715-0.929)	0.792 (0.689-0.895)	0.879 (0.790-0.967)
Accuracy, %	84 (75-93)	81 (73-90)	86 (78-93)
Sensitivity, %	79 (59-95)	74 (55-91)	80 (63-95)
Specificity, %	85 (75-95)	84 (75-93)	88 (79-95)
PPV, %	68 (47-87)	65 (46-83)	74 (57-90)
NPV, %	91 (82-98)	89 (80-96)	91 (83-98)

Values are n (%) or AUC (95% CI). The 2 guidelines classified different patients as indeterminate; therefore, their performance was evaluated in overlapping but nonidentical subsets after excluding indeterminate cases: the 2016 ASE/EACVI guidelines were evaluated in 67/83 (81%) patients, whereas the 2025 ASE guidelines were evaluated in 81/83 (98%) patients. The DL model was evaluated in all patients (n = 83). To enable the straightforward calculation of diagnostic performance metrics (ie, accuracy, sensitivity, specificity, positive predictive value, and negative predictive value), both guideline-based classifications were dichotomized by merging the “normal diastolic function” and the “DD with normal LAP” groups (as both are supposed to have normal LAP). The DL-predicted probabilities were dichotomized using a cutoff of 0.99957, optimized in this cohort by maximizing Youden J statistic.

AUC = area under the receiver operating characteristic curve, NPV = negative predictive value, PPV = positive predictive value; other abbreviations as in Table 1.

Of the 130 patients of the second validation cohort, 30 (23%) had elevated LVFP based on invasive hemodynamic assessment. Fifty (38%) patients underwent echocardiography and RHC on the same day, whereas the other 80 (62%) underwent both tests within 24 hours. The clinical and echocardiographic characteristics of this cohort are reported in Table 1 and Supplemental Table 14. The Sankey diagram showed notable reclassification in this cohort as well (Figure 2). The 2025 guidelines classified significantly fewer patients as indeterminate than the 2016 guidelines (12/130 [9%] vs 39/130 [30%]; *P* < 0.001). The DL-predicted probability detected elevated LVFP with an AUC of 0.816 (95% CI: 0.730-0.903), which was significantly higher than the AUCs of the 2016 (0.634 [95% CI: 0.533-0.735]; *P* < 0.001) and the 2025 guidelines (0.694 [95% CI: 0.596-0.791]; *P* = 0.004) (Figure 5A). The discriminatory power of the 2016 and 2025 guidelines was similar (*P* = 0.209). The DL model also demonstrated superior performance compared with both guidelines when the indeterminate groups (Figure 5B, Table 5), patients with atrial fibrillation (Supplemental Figure 4), or those with both LARS and IVRT missing (Supplemental Figure 5) were excluded from the analysis.

**Table 5:** Performance of the Guidelines and the DL Model in Detecting Elevated LVFP in the Second Hemodynamic Validation Cohort (Indeterminate Groups Excluded)

	2016 ASE/EACVI Guidelines	2025 ASE Guidelines	DL Model
Indeterminate diastolic function or DD with indeterminate LAP	39 (30)	12 (9)	—
AUC	0.607 (0.486-0.727)	0.697 (0.597-0.797)	0.816 (0.730-0.903)
Accuracy, %	68 (59-77)	68 (59-76)	76 (68-83)
Sensitivity, %	48 (26-70)	73 (55-89)	70 (53-86)
Specificity, %	74 (64-83)	66 (57-76)	78 (70-86)
PPV, %	33 (17-52)	38 (25-52)	49 (34-64)
NPV, %	84 (74-92)	90 (82-96)	90 (83-96)

Values are n (%) or AUC (95% CI). The 2 guidelines classified different patients as indeterminate; therefore, their performance was evaluated in overlapping but nonidentical subsets after excluding indeterminate cases: the 2016 ASE/EACVI guidelines were evaluated in 91/130 (70%) patients, whereas the 2025 ASE guidelines were evaluated in 118/130 (91%) patients. The DL model was evaluated in all patients (n = 130). To enable the straightforward calculation of diagnostic performance metrics (ie, accuracy, sensitivity, specificity, positive predictive value, and negative predictive value), both guideline-based classifications were dichotomized by merging the “normal diastolic function” and the “DD with normal LAP” groups (as both are supposed to have normal LAP). To preserve the operating point derived in the first hemodynamic validation cohort while avoiding cohort-specific optimization, percentile-based operating-point transport was applied to this cohort. Accordingly, the top 32.5% of DL-predicted probabilities were classified as positive, corresponding to a cohort-specific numeric cutoff of 0.98612.

Abbreviations as in Tables 1 and 4.

## Discussion

In this extensive comparative study, we evaluated the performance of the 2025 ASE guidelines, the 2016 ASE/EACVI guidelines, and a validated DL model for assessing diastolic function (Central Illustration). We leveraged the racially and demographically diverse ARIC cohort from multiple communities across the United States and 2 geographically distinct hemodynamic validation cohorts with invasively measured LVFP to evaluate the generalizability and robustness of these 3 approaches across various populations and settings. Importantly, these cohorts were derived from well-adjudicated clinical registries and reflect real-world clinical settings in which complete acquisition of Doppler and strain parameters is not uniformly achievable, resulting in the absence of ≥1 guideline-recommended parameters in a substantial proportion of patients. Although the 2025 guidelines markedly reduced indeterminate classifications compared with the 2016 guidelines, the DL model yielded better prognostic discrimination than both guidelines in the ARIC cohort and also outperformed the H_2_FPEF score among patients with preserved LVEF. Furthermore, the DL model demonstrated superior or similar performance in detecting elevated LVFP in the 2 hemodynamic validation cohorts. Notably, this performance was achieved using routinely acquired echocardiographic variables, without reliance on the full complement of advanced parameters.

**Central Illustration:**
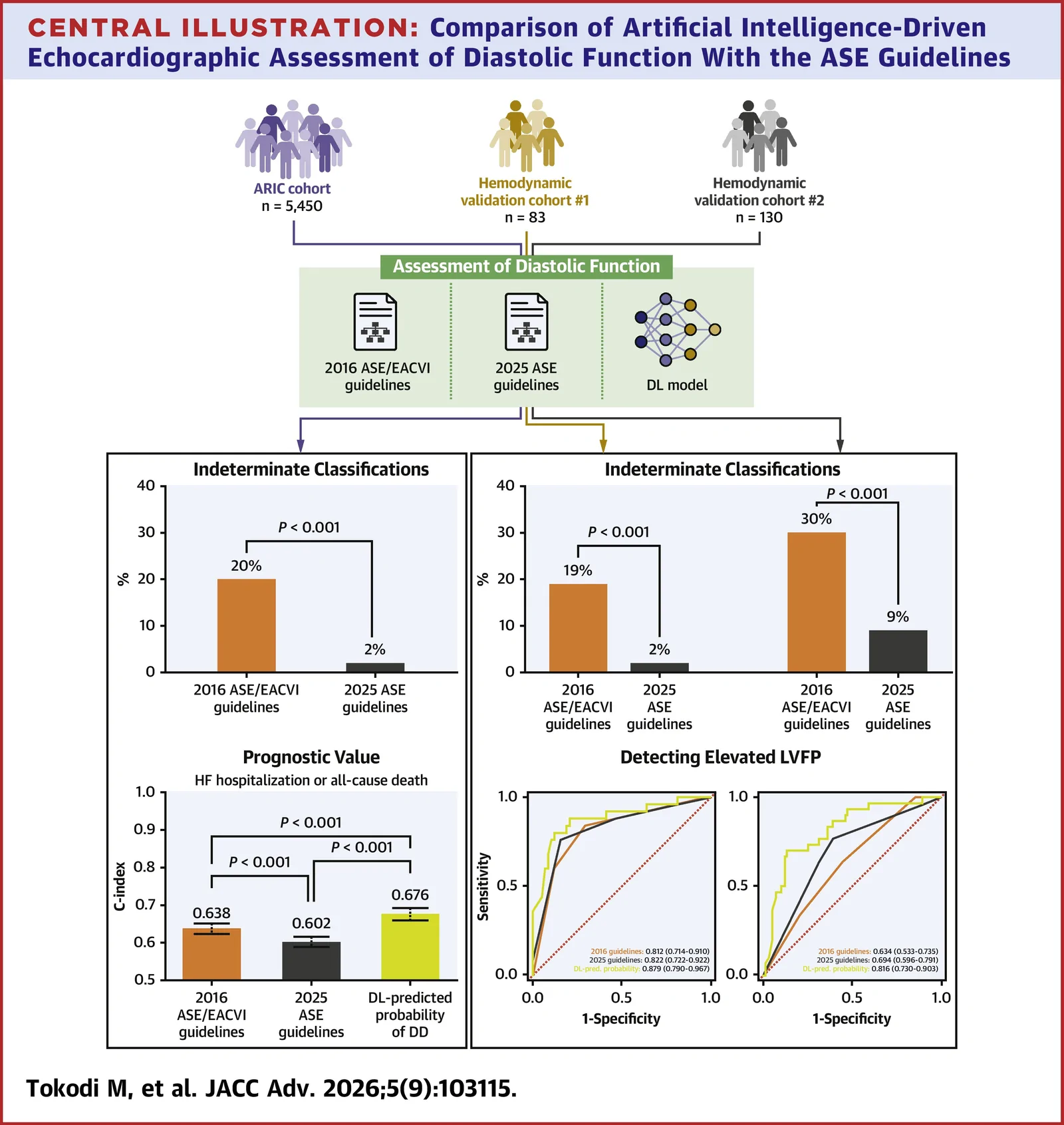
Comparison of Artificial Intelligence-Driven Echocardiographic Assessment of Diastolic Function With the ASE Guidelines In this study, we compared the prognostic and diagnostic performance of the newly released 2025 ASE guidelines, the earlier 2016 ASE/EACVI guidelines, and a rigorously validated DL model for assessing LV diastolic function. Although the 2025 guidelines markedly reduced indeterminate classifications in all 3 cohorts compared with the 2016 guidelines, they demonstrated lower prognostic value and similar diagnostic performance for detecting elevated LVFP in the hemodynamic validation cohorts. The DL model yielded better prognostic discrimination than both guidelines in the ARIC cohort and showed superior performance in detecting elevated LVFP. Nevertheless, because ≥1 guideline-recommended parameters were unavailable in a substantial proportion of patients in the analyzed cohorts, these findings should be regarded as exploratory pending validation in data sets with complete guideline-recommended data. ARIC = Atherosclerosis Risk In Communities; ASE = American Society of Echocardiography; DD = diastolic dysfunction; DL = deep learning; EACVI = European Association of Cardiovascular Imaging; HF = heart failure; LV = left ventricular; LVFP = left ventricular filling pressure.

### Comparison of the 2025 and 2016 guidelines

The 2016 ASE/EACVI guidelines were designed for broad clinical usability but were limited by high rates of indeterminate classifications and their moderate correlation with invasive measures of LVFP.^7^, ^8^, ^9^, ^10^, ^11^ The 2025 ASE guidelines introduced several notable refinements and improvements, such as the incorporation of additional echocardiographic features (eg, LARS) and the use of age-specific cutoff values.^5^ They also substantially reduced the proportion of indeterminate cases, as demonstrated in recent studies.^12^^,^^29^^,^^30^ Consistent with these prior findings, we observed a marked reduction in indeterminate classifications, from 20% to 2% in the ARIC cohort, from 19% to 2% in the first hemodynamic validation cohort, and from 30% to 9% in the second hemodynamic validation cohort, despite missing pulmonary vein systolic and diastolic velocities (S/D) and limited availability of LARS, reflecting real-world variability in data acquisition.

Nevertheless, despite the advantages noted previously, the 2025 guidelines did not surpass the 2016 guidelines in prognostic or diagnostic performance in our analyses. These findings contrast with those of Lababidi et al,^12^ likely reflecting differences in patient selection (eg, exclusion of patients with persistent or permanent atrial fibrillation) and analytic approach. Specifically, they used the McNemar test to compare paired classification accuracies between the guidelines, which required excluding all cases classified as indeterminate by either guideline version.^12^ Although this approach emphasizes performance in definitively classifiable patients, it may overlook the real-world clinical burden of indeterminate classifications. Therefore, we reported 2 sets of performance metrics: one that included all patients, with indeterminate results treated as nonconclusive, and another that excluded indeterminate cases separately for each guideline version. Importantly, our findings align with those of Harada et al,^13^ who reported limited discriminative performance of the 2025 guidelines despite fewer indeterminate cases. Similarly, Hafez et al,^29^ also observed no improvement in prognostic discrimination with the 2025 guidelines compared with the 2016 guidelines in a large patient cohort. Hu et al,^30^ likewise reported similar diagnostic accuracies for the 2 guideline versions in detecting elevated LVFP in a prospective cohort of patients undergoing ablation for paroxysmal atrial fibrillation. Taken together, these results suggest that improvements in classification do not necessarily translate into higher accuracy, highlighting limitations of rule-based approaches.

### Comparison of the DL model and expert guidelines

Across the 3 analyzed cohorts, the DL model demonstrated higher prognostic discrimination than both the 2016 ASE/EACVI and 2025 ASE guidelines and comparable or superior diagnostic performance for detecting elevated LVFP. Moreover, the DL model maintained its performance across multiple sensitivity analyses and patient subgroups, supporting the robustness and potential clinical utility of its predictions in diverse patient populations and practice settings. However, a critical consideration is that these comparisons were performed in data sets where ≥1 guideline-recommended parameters were unavailable in a substantial proportion of patients. Therefore, the present analysis reflects guideline application under real-world data constraints rather than a definitive comparison against fully implemented guideline assessments.

It is also important to acknowledge that the DL model, by producing a continuous probabilistic output, has an inherent structural advantage over guideline-based assessments that classify patients into discrete categories. Accordingly, these comparisons should not be interpreted as a symmetric head-to-head evaluation of equivalent tools, and the higher C-index achieved by the DL model may partly reflect this fundamental difference in output structure rather than clinical superiority alone, even though the observed performance advantage persisted even after dichotomization of the DL model’s outputs. Furthermore, the DL model was intentionally designed to accommodate missing data, whereas the guideline algorithms were applied without their full recommended parameter set. These asymmetries between the DL model and the guideline-based approaches should be taken into consideration when interpreting the results of the present study.

Another important consideration relates to the comparison in the first hemodynamic validation cohort, where the lack of a significant difference in AUC between the DL model and the 2016 guidelines when indeterminate classifications were included may appear discrepant from our previously reported findings in the same cohort.^15^ Importantly, this apparent discrepancy arises from the use of different statistical approaches for ROC analysis. In our previous study, the 2016 guideline-based classifications were treated as an ordinal variable, with the 3 categories assigned ranks of 0 (DD with indeterminate LAP), 1 (normal diastolic function or grade 1 DD), and 2 (grade 2 or 3 DD) on an information scale, where 0 denoted the absence of conclusive diagnostic information regarding LVFP, and these ranks were then used directly as predictors in the ROC analysis.^15^ This approach implicitly assumed a monotonic ordering and a linear increase in information content across categories, similar to the Lung-RADS^31^ or the BI-RADS^32^ classification systems. However, subsequent studies have shown that patients with indeterminate diastolic function have a worse prognosis than those with no DD,^33^ suggesting that indeterminate classifications may carry clinically meaningful information rather than representing a complete absence of information. To account for these observations, in the present study, we modified our previous approach and applied logistic regression that treated the guideline-based classifications as nominal variables, and we used the resulting predicted probabilities for ROC analysis. Consequently, the 2 ROC analyses are based on different statistical representations of the guideline classifications necessitated by new domain knowledge and are therefore not directly comparable.

### Clinical implications and future directions

The findings have several clinical implications. First, HF with preserved ejection fraction diagnosis remains challenging and often requires invasive hemodynamic examinations, leading to delays and missed opportunities. A probabilistic, data-driven model may provide a more individualized assessment than rule-based algorithms and enable earlier identification of high-risk patients. Second, the emergence of several new HF with preserved ejection fraction therapies, including sodium-glucose cotransporter-2 inhibitors,^34^ glucagon-like peptide-1 receptor agonists,^35^ and finerenone,^36^ underscores the importance of early diagnosis, as improved risk stratification may facilitate timely initiation of these disease-modifying therapies and improve outcomes. Third, the DL model may support clinical trials and longitudinal research by enabling standardized phenotyping. Fourth, its compatibility with routinely acquired echocardiographic parameters makes the model well-suited for real-world implementation through integration into existing reporting software solutions or electronic health record systems to assist clinical decision-making. Nevertheless, before clinical deployment, site-specific validation would be required, as model performance may vary according to patient characteristics, imaging equipment, and acquisition practices.

### Study limitations

Despite its strengths, the present study has several limitations. First, excluding patients with more than 3 missing values among the DL model’s input features may have introduced selection bias. However, because the same parameters are also required by the guideline algorithms, this exclusion criterion was applied equally across all 3 approaches and is therefore unlikely to explain the observed differences in performance. Second, some parameters recommended in the 2025 guidelines (eg, LARS, IVRT, pulmonary vein S/D) were not consistently available in the analyzed cohorts. Notably, other recent studies evaluating the 2025 ASE guidelines similarly lacked pulmonary vein Doppler signals due to feasibility constraints.^13^^,^^29^ Furthermore, even in the initial validation study by Lababidi et al,^12^ pulmonary vein S/D and atrial velocity duration were feasible in only 48% and 38% of cases, respectively. Therefore, missing data reflect real-world practice rather than a study-specific limitation. Third, prospective implementation of the DL model remains to be established in terms of patient outcomes, workflow efficiency, or treatment decisions. Integration into mobile and cloud-based platforms could help bridge this gap by facilitating prospective implementation and enabling remote HF screening and triage, particularly in resource-limited settings. Fourth, despite previous foundational work showing that the DL model can identify transparent phenogroups,^15^ clinicians may still perceive it as a “black box”. Lastly, the hemodynamic validation cohorts were relatively small, represented selected patient populations, and included only resting hemodynamic measurements. Therefore, further validation in larger and more diverse cohorts is needed to establish the generalizability of our findings.

## Conclusions

In the present analyses, where not all guideline-recommended parameters were available, the DL model demonstrated the potential to improve prognostic and diagnostic performance compared with both the 2025 ASE and 2016 ASE/EACVI guidelines. The DL model achieved this performance using only routinely acquired echocardiographic variables, highlighting the ability of data-driven approaches to capture clinically relevant physiologic information in real-world settings where the acquisition of advanced parameters may not be consistently feasible. However, these findings should be considered exploratory and require validation in datasets with complete guideline-recommended data to determine whether the observed advantages persist under conditions of comprehensive guideline implementation.

## Funding Support and Author Disclosures

Dr Tokodi has been supported by the János Bolyai Research Scholarship of the Hungarian Academy of Sciences; and has received grant support from the National Research, Development and Innovation Office (NKFIH) of Hungary (STARTING 152450) and through the MILAB project (Project No. RRF-2.3.1-21-2022-00004), funded by the European Union. Dr Kagiyama received research grants from AstraZeneca, Bristol Myers Squibb, EchoNous Inc, and AMI Inc; speaker honoraria from Eli Lilly, Novartis, Otsuka Pharmaceutical, Bristol Myers Squibb, and Boehringer Ingelheim; and was affiliated with a department funded by Paramount Bed Ltd. Dr Pandey is a consultant for Tricog, MedicalAI, Anumana, and Ultromics. Dr Lam has received research grants from the National Medical Research Council of Singapore, Novo Nordisk, and Roche Diagnostics; has served in advisory, consulting, and trial leadership roles for Alnylam Pharma, AnaCardio AB, Applied Therapeutics, AstraZeneca, Bayer, Boehringer Ingelheim, Boston Scientific, BridgeBio, Corteria, Corveus Medical, CPC Clinical Research, Cytokinetics, Daiichi Sankyo, Eli Lilly, EvlaBio, ICON Clinical Research, Impulse Dynamics, Intellia Therapeutics, Janssen Research & Development LLC, Klyv Therapeutics, Medscape/WebMD Global LLC, Merck, Novartis, Novo Nordisk, Pfizer, Radcliffe Group, Ribocure, Roche, Tenax, and Us2.ai; has patent application PCT/SG2016/050217 pending and holds several United States patents (10,702,247 B2; 10,631,828 B1; 11,301,996 B2; 11,446,009 B2; 11,931,207 B2; 12,001,939; and 12,400,762 B2); and is a co-founder and non-executive director of Us2.ai. Dr Yanamala has received grants or contracts from MindMics, RCE Technologies, HeartSciences, and Abiomed; consulting fees from Turnkey Learning and Turnkey Insights; honoraria or travel support from West Virginia University and the National Science Foundation; she is an advisor to Research Spark Hub and Magnetic 3D; a faculty member at Carnegie Mellon University; an editorial board member of the American Society of Echocardiography; and a special government employee at the FDA Center for Devices and Radiological Health. Dr Sengupta is supported by grants from the National Institutes of Health/National Heart, Lung, and Blood Institute (1R01HL173998-01A1, 3U01HL088942-17S, 1P50MD017356-01) and National Science Foundation (#2125872); he has served on the Advisory Board of RCE Technologies and HeartSciences and holds stock options; received grants or contracts from RCE Technologies, HeartSciences, Butterfly, and MindMics; and holds patents with Mayo Clinic (US8328724B2), HeartSciences (US11445918B2), and Rutgers Health (62/864,771; US202163152686P; WO2022182603A1; US202163211829P; WO2022266288A1; US202163212228P). The deep learning model used in this study is owned by Rutgers University, managed through the Rutgers Office for Research and its Technology Transfer program, and has been licensed to Us2.ai. All other authors have reported that they have no relationships relevant to the contents of this paper to disclose.

## Appendix

Supplemental Methods and References ad Supplemental Tables 1 to 14 and Supplemental Figures 1 to 5
