# Supplementary material for: Comparison of Artificial Intelligence-Driven Echocardiographic Assessment of Diastolic Function With the ASE Guidelines
Source: JACC Adv. 2026 Sep 23;5(9):103115. doi: 10.1016/j.jacadv.2026.103115 (PMC13616516; doi:10.1016/j.jacadv.2026.103115)
Supplement: Supplementary file 1 [file mmc1.pdf]

SUPPLEMENTARY APPENDIX

Comparison of Artificial Intelligence-Driven Echocardiographic Assessment of Diastolic Function With the ASE Guidelines

SUPPLEMENTAL METHODS

Echocardiographic protocol in the ARIC cohort study

Echocardiographic examinations were performed by experienced echocardiographers at each field center using commercially available ultrasound systems (iE33, Philips Medical Systems with Vision 2011). The protocol included 2D, color Doppler, spectral Doppler, and tissue Doppler imaging. All measurements and evaluations were conducted at the Atherosclerosis Risk in Communities (ARIC) Echocardiography Reading Center (Brigham and Women's Cardiac Imaging Core Laboratory, Boston, MA). All analyses were over-read (blinded to study participants' clinical information) and were approved by board-certified echocardiographers.

Calculation of the H_2FPEF score

H_2FPEF score was calculated according to the original published algorithm (1), which incorporates the following six clinical and echocardiographic variables: body mass index >30 kg/m² (2 points); use of ≥2 antihypertensive medications (1 point); paroxysmal or persistent atrial fibrillation (3 points); Doppler echocardiography-estimated pulmonary artery systolic pressure >35 mmHg (1 point); age >60 years (1 point); and ratio of early mitral inflow velocity and early diastolic mitral annular velocity at septal position (E/e') >9 (1 point). Each component was assigned a point value based on the original weighting scheme, resulting in total scores ranging from 0 to 9, with higher scores indicating a greater likelihood of heart failure with preserved ejection fraction (EF).

Development of the DL model for assessing diastolic function

To develop a deep learning (DL) model for assessing diastolic function, topological data analysis (TDA) was first performed using the Ayasdi Workbench (version 7.9, Ayasdi Inc., Palo Alto, California) to simultaneously evaluate nine echocardiographic features (i.e., left ventricular mass index [LVMi], left ventricular EF [LVEF], early mitral inflow velocity [E], late mitral inflow velocity [A], E/A, septal e', septal E/e', left atrial volume index [LAVi], and tricuspid regurgitation peak velocity [TRV]) of 1,242 patients with varying degrees of systolic and diastolic dysfunction (2,3). TDA applies the tools of shape analysis to identify and connect highly similar data points (e.g., patients) in a multi-dimensional space and then plots the identified connections as a two-dimensional similarity network (4). The generated network consists of nodes (representing collections of similar data points) connected by edges (i.e., lines between two nodes) if they have at least one data point in common. In the analyzed dataset, TDA yielded a circular similarity network that was divided into two parts based on the differences in clinical characteristics and outcomes of patients located in its different regions: one comprising patients with low risk and the other encompassing patients with a high risk of diastolic dysfunction (DD). These labels were assigned to each of the 1,242 patients in the similarity network and were used to train classifiers in a subsequent supervised learning phase, enabling the assessment of diastolic function in new, previously unseen patients.

For training and evaluation of the classifiers, BigML (BigML Inc., Corvallis, OR, http://bigml.com), a cloud-based machine learning platform, was utilized. After splitting the cohort into training and test sets in an 80:20 ratio, various classifiers (i.e., decision trees, ensembles of decision trees, logistic regression models, and deep neural networks) were trained on the training set (with cross-validation) and subsequently evaluated on the test set. Missing values were handled natively by the learning algorithm: missing values in categorical features were treated as a distinct input category, while missing numeric values were explicitly modeled

rather than imputed or excluded, both during training and inference, using BigML's default configuration. Model selection and hyperparameter tuning were performed using the OptiML optimization process, which leverages Bayesian optimization to evaluate hundreds of supervised models and identify the best-performing ones (5). These models can then be used individually or combined into a so-called *fusion model*.

The final DL model is a *fusion model* that combines the seven best-performing deep neural networks and averages their predictions (3). In this model, the most important feature for predicting DD is e', followed by E/e', LVMi, LVEF, LAVi, TRV, E, A, and E/A in decreasing order of importance. The model has been externally validated in multiple cohorts, showing that higher DL-predicted DD probability values were associated with elevated LV filling pressures (LVFP), increased neurohormonal activation, lower exercise capacity, and a higher incidence of adverse cardiac events during follow-up (3). The DL model is publicly available at https://wvu-model.herokuapp.com. A more detailed description of model development and evaluation has been published previously (2,3,6).

Dichotomizing the output of the DL model

To verify that the DL model's superior performance over the guidelines was not solely attributable to its ability to generate continuous risk estimates, in contrast to the categorical nature of the guideline-based classifications, C-indices and areas under the receiver operating characteristic curve (AUCs) were also calculated and compared after dichotomizing the DL model's output. In the ARIC cohort, the default 0.5 threshold was used to dichotomize the DL-predicted probabilities. Given that the DL model was primarily trained for prognostication, a cutoff optimized for identifying elevated LVFP was applied instead of the default 0.5 threshold to dichotomize the predicted probabilities in the hemodynamic validation cohorts. In the first hemodynamic validation cohort, the classification threshold was selected by maximizing

Youden’s J statistic, yielding an optimal cutoff of 0.99957 and a predicted-positive rate of 32.5%. To preserve the operating point derived in the first cohort while avoiding cohort-specific optimization, percentile-based operating-point transport was applied to the second hemodynamic validation cohort (7). Accordingly, the top 32.5% of DL-predicted probabilities were classified as positive in that cohort, corresponding to a cohort-specific numeric cutoff of 0.98612. To ensure a fair comparison, the outputs of both guideline-based classifications were also dichotomized by combining all categories except for “DD with elevated LAP”.

SUPPLEMENTAL RESULTS

Results using the dichotomized output of the DL model

The DL model demonstrated superior prognostic power over both guidelines in univariable Cox regression even when its predicted probabilities were dichtomized, with a C-index of 0.627 (95% CI: 0.614–0.640) vs. 0.543 (95% CI: 0.534–0.552) for the 2016 guidelines and 0.556 (95% CI: 0.546–0.566) for the 2025 guidelines ($p<0.001$ for both comparisons).

In the first hemodynamic validation cohort, the dichotomized output of the DL model exhibited an AUC of 0.840 (95% CI: 0.749–0.930) in identifying patients with elevated LVFP, which was higher than the AUC of the 2025 guidelines (0.728 [95% CI: 0.621–0.835], $p=0.041$) and was numerically higher than that of the 2016 guidelines, although the latter difference did not reach statistical significance (0.740 [95% CI: 0.633–0.846], $p=0.066$).

In the second hemodynamic validation cohort, the dichotomized output of the DL model detected elevated LVFP with an AUC of 0.785 (95% CI: 0.695–0.875), which was significantly higher than the AUCs of the 2016 (0.567 [95% CI: 0.472–0.661], $p<0.001$) and the 2025 guidelines (0.662 [95% CI: 0.563–0.761], $p=0.012$).

REFERENCES

1. Reddy YNV, Carter RE, Obokata M, Redfield MM, Borlaug BA. A Simple, Evidence-Based Approach to Help Guide Diagnosis of Heart Failure With Preserved Ejection Fraction. Circulation 2018;138:861-870.
2. Tokodi M, Shrestha S, Bianco C et al. Interpatient Similarities in Cardiac Function: A Platform for Personalized Cardiovascular Medicine. JACC Cardiovasc Imaging 2020;13:1119-1132.
3. Pandey A, Kagiyama N, Yanamala N et al. Deep-Learning Models for the Echocardiographic Assessment of Diastolic Dysfunction. JACC Cardiovasc Imaging 2021;14:1887-1900.
4. Lum PY, Singh G, Lehman A et al. Extracting insights from the shape of complex data using topology. Scientific Reports 2013;3:1236.
5. Shahriari B, Swersky K, Wang Z, Adams RP, Freitas Nd. Taking the Human Out of the Loop: A Review of Bayesian Optimization. Proceedings of the IEEE 2016;104:148-175.
6. Tokodi M, Shah R, Jamthikar A et al. Deep Learning Model of Diastolic Dysfunction Risk Stratifies the Progression of Early-Stage Aortic Stenosis. JACC Cardiovasc Imaging 2025;18:150-165.
7. Obuchowski NA. Receiver operating characteristic curves and their use in radiology. Radiology 2003;229:3-8.

Supplemental Table 1. Availability of the echocardiographic parameters used for LV diastolic function assessment in the three cohorts

	ARIC cohort	Hemodynamic validation cohort #1	Hemodynamic validation cohort #2
**LVEF**	97	100	100
**LVMi**	99	94	98
**E**	100	100	100
**E-DT**	100	99	99
**A**	96	94	83
**E/A**	96	94	83
**IVRT**	0	75	42
**e' (septal)**	100	100	100
**E/e' (septal)**	100	100	100
**e' (lateral)**	100	98	60
**E/e' (lateral)**	100	98	60
**e' (average)**	100	98	60
**E/e' (average)**	100	98	60
**LAVi**	99	96	83
**LARS**	0	78	95
**TRV**	58	53	96
**PASP**	0	37	52
**Pulmonary vein S/D**	97	100	100

Cell values are the percentages denoting the availability of each echocardiographic parameter. Cells are color-coded based on the percentage value, with green indicating high availability and red indicating low availability.

A – late mitral inflow velocity, ARIC – Atherosclerosis Risk in Communities study, D – pulmonary vein diastolic velocity, E – early mitral inflow velocity, E-DT – E-wave deceleration time, e' – early diastolic mitral annular velocity, IVRT – isovolumic relaxation time, LARS – left atrial reservoir strain, LAVi – left atrial volume index, LV – left ventricular, LVEF – left ventricular ejection fraction, LVMi – left ventricular mass index, PASP – pulmonary artery systolic pressure estimated by echocardiography, S – pulmonary vein systolic velocity, TRV – tricuspid regurgitation peak velocity

Supplemental Table 2. Proposed Requirements for Cardiovascular Imaging-Related Multimodal-AI Evaluation (PRIME 2.0) checklist

Checklist item	Manuscript section/figure/table
**1. Designing an AI study in cardiovascular imaging**	
1.1 Appropriateness of applying AI	
Describe the need for applying AI	Introduction
Determine the appropriateness of applying AI	Introduction
1.2 Study objectives, input data type, and prediction target	
Explain the AI task and the likely deployment context	Introduction<br>Discussion<br>Also explained previously in the paper reporting the development and initial validation of the DL model:<br>https://doi.org/10.1016/j.jcmg.2021.04.010
Describe the input data, number of training/test examples	Methods – Validation cohort for predicting clinical outcomes<br>Methods – Validation cohorts for detecting elevated LVFP<br>Figure 1, Table 1<br>Supplemental Methods – Development of the DL model for assessing diastolic function<br>Also described previously in the paper reporting the development and initial validation of the DL model:<br>https://doi.org/10.1016/j.jcmg.2021.04.010
Specify model supervision type	Methods – DL-based assessment of diastolic function<br>Supplemental Methods – Development of the DL model for assessing diastolic function<br>Discussion – Comparison of the DL model and expert guidelines<br>Also described previously in the paper reporting the development and initial validation of the DL model:<br>https://doi.org/10.1016/j.jcmg.2021.04.010
Describe the nature of the model's output and what it represents	Methods – DL-based assessment of diastolic function<br>Supplemental Methods – Development of the DL model for assessing diastolic function<br>Also described previously in the paper reporting the development and initial validation of the DL model:<br>https://doi.org/10.1016/j.jcmg.2021.04.010
1.3 Design of the AI study	
Describe the study design	External validation of a pre-existing DL model using data previously collected in prospective studies and registries.<br>Central Illustration

Checklist item	Manuscript section/figure/table
Describe data origin	Methods – Validation cohort for predicting clinical outcomes<br>Methods – Validation cohorts for detecting elevated LVFP
Describe whether impact analysis was included	Impact analysis was not performed.
**2. Data format and preprocessing**	
2.1 Data format	
Describe the technical details of the data acquisition	Methods – Validation cohort for predicting clinical outcomes<br>Methods – Validation cohorts for detecting elevated LVFP<br>Supplemental Methods – Echocardiographic protocol in the ARIC cohort study<br>Also described previously in the paper reporting the development and initial validation of the DL model: https://doi.org/10.1016/j.jcmg.2021.04.010
Describe the technical details of the data format	Only tabular data was used in this study.
2.2 Clinical characteristics of the study cohort	
Present the age, sex, and race distributions of the cohorts	Table 1<br>Supplemental Tables 3, 13, and 14
Summarize key clinical and imaging characteristics of the cohorts	Table 1<br>Supplemental Tables 3, 13, and 14
Compare summary statistics of cases and controls	Supplemental Tables 3, 13, and 14
2.3 Steps of data preprocessing	
Describe how data were cleaned, made uniform, and consistent	Methods – Validation cohort for predicting clinical outcomes<br>Methods – Validation cohorts for detecting elevated LVFP<br>Supplemental Methods – Echocardiographic protocol in the ARIC cohort study<br>Also described previously in the paper reporting the development and initial validation of the DL model: https://doi.org/10.1016/j.jcmg.2021.04.010
Describe data harmonization techniques (if applicable)	N/A
Provide details on missing values and imputation methods	Supplemental Tables 1, 3, 4, 13, and 14
Describe processes for handling outliers	Supplemental Methods – Development of the DL model for assessing diastolic function<br>Also described previously in the paper reporting the development and initial validation of the DL model: https://doi.org/10.1016/j.jcmg.2021.04.010

Checklist item	Manuscript section/figure/table
Describe whether class imbalance exists	Results – Prognostic value in the entire ARIC cohort<br>Results – Prognostic value in ARIC study participants with preserved LVEF<br>Results – Performance in detecting elevated LVFP
2.4 Feature engineering and feature selection	
Describe applied feature engineering techniques	N/A
Describe applied feature selection techniques	Described previously in the paper reporting the development and initial validation of the DL model: https://doi.org/10.1016/j.jcmg.2021.04.010
**3. Selection of AI methods and applications**	
3.1 Selecting appropriate AI methods and applications	
Clearly define data composition (structured/unstructured)	Supplemental Methods – Development of the DL model for assessing diastolic function<br>Also described previously in the paper reporting the development and initial validation of the DL model: https://doi.org/10.1016/j.jcmg.2021.04.010
3.2–3.4 Training strategies	
Describe the AI method used, with rationale for clinical task fit	Supplemental Methods – Development of the DL model for assessing diastolic function<br>Also described previously in the paper reporting the development and initial validation of the DL model: https://doi.org/10.1016/j.jcmg.2021.04.010
3.5 Solving clinical problems	Supplemental Methods – Development of the DL model for assessing diastolic function<br>Also described previously in the paper reporting the development and initial validation of the DL model: https://doi.org/10.1016/j.jcmg.2021.04.010
**4. Model assessment**	
4.1 Importance of model assessment	
Describe the evaluation approach and how it addresses the clinical question	Methods – Statistical analysis<br>Supplemental Methods – Development of the DL model for assessing diastolic function<br>Also described previously in the paper reporting the development and initial validation of the DL model: https://doi.org/10.1016/j.jcmg.2021.04.010

Checklist item	Manuscript section/figure/table
4.2 Technical performance metrics	
Report relevant performance metrics	Results – Prognostic value in the entire ARIC cohort<br>Results – Prognostic value in ARIC study participants with preserved LVEF<br>Results – Performance in detecting elevated LVFP<br>Tables 3, 4, and 5<br>Figures 3 and 5<br>Supplemental Tables 6, 7, and 8<br>Supplemental Figures 1, 2, 3, 4, and 5
Justify metric selection based on task characteristics	Methods – Statistical analysis
Describe manual annotation process, reference-standard prep, and observer variability	Methods – Validation cohort for predicting clinical outcomes<br>Methods – Validation cohorts for detecting elevated LVFP<br>Supplemental Methods – Echocardiographic protocol in the ARIC cohort study
4.3–4.4 Robustness, generalizability, and evaluating data quality	
Evaluate model robustness to external variations	Results – Prognostic value in the entire ARIC cohort<br>Results – Prognostic value in ARIC study participants with preserved LVEF<br>Results – Performance in detecting elevated LVFP<br>Tables 3, 4, and 5<br>Figures 3 and 5<br>Supplemental Tables 6, 7, and 8<br>Supplemental Figures 1, 2, 3, 4, and 5<br>Also assessed previously in these papers:<br>https://doi.org/10.1016/j.jcmg.2021.04.010<br>https://doi.org/10.1016/j.jcmg.2024.07.017<br>https://doi.org/10.1093/ehjci/jeae037
Assess performance across clinical subpopulations and sociodemographic subgroups	
4.5 Identifying features learned by the model	
Describe interpretability/explainability methods	Supplemental Methods – Development of the DL model for assessing diastolic function<br>Also described previously in the paper reporting the development and initial validation of the DL model:<br>https://doi.org/10.1016/j.jcmg.2021.04.010
Quantify and discuss uncertainty	N/A
**5. Clinical evaluation**	
5.1 Importance of clinical evaluation	
Describe potential clinical impact of misclassification	Misclassification could lead to delayed diagnosis, inappropriate risk stratification, and suboptimal patient management.

Checklist item	Manuscript section/figure/table
5.2 Clinical utility metrics	
Define clinical utility of the AI system	Results – Prognostic value in the entire ARIC cohort<br>Results – Prognostic value in ARIC study participants with preserved LVEF<br>Results – Performance in detecting elevated LVFP<br>Tables 3, 4, and 5, Figures 3 and 5<br>Supplemental Tables 6, 7, and 8<br>Supplemental Figures 1, 2, 3, 4, and 5<br>Discussion – Clinical implications and future directions
Describe cost-effectiveness analysis	Cost-effectiveness analysis was not performed.
5.3 Clinical validation	
Provide evidence of clinical validation	Results – Prognostic value in the entire ARIC cohort<br>Results – Prognostic value in ARIC study participants with preserved LVEF<br>Results – Performance in detecting elevated LVFP<br>Tables 3, 4, and 5, Figures 3 and 5<br>Supplemental Tables 6, 7, and 8<br>Supplemental Figures 1, 2, 3, 4, and 5
5.4 Continuous monitoring	
Outline plans for post-deployment monitoring	The DL model's performance will be assessed in additional validation cohorts. If performance degradation is detected, the precise cause will be identified, and the model will be refined (e.g., by updating training data [retraining], model parameter values [recalibrating], or improving the modeling structure or training method).
**6. Best practices for replicability**	
6.1 Importance of transparency and open science principles	
Ensure data sharing follows FAIR (findability, accessibility, interoperability, and reusability principles	Data could not be shared due to proprietary and institutional restrictions. Access to the ARIC dataset can be requested at the following website: https://biolincc.nhlbi.nih.gov/studies/aric/
Report on training data representativeness	Reported previously in the paper describing the development and initial validation of the DL model: https://doi.org/10.1016/j.jcmg.2021.04.010
6.2 Ensuring technical reproducibility	
Report results for the final model and training process	Supplemental Methods – Development of the DL model for assessing diastolic function<br>Also reported previously in the paper describing the development and initial validation of the DL model: https://doi.org/10.1016/j.jcmg.2021.04.010
Describe sources of randomness in training	
Report random seeds (if applicable)	
Describe hardware setup	
Evaluate uncertainty from randomness	

Checklist item	Manuscript section/figure/table
Justify if the source code, model weights, or datasets are not shared	Although these are not publicly available due to proprietary and institutional restrictions, the DL model is accessible online: https://wvu-model.herokuapp.com
6.3 Specific considerations for reproducing large language model and generative AI studies	N/A
**7. Reporting of limitations, biases, and alternatives**	
7.1 Acknowledging study and model limitations	
Discuss key limitations	Discussion – Limitations
Report sensitivity analyses and model-specific issues	Results – Prognostic value in the entire ARIC cohort<br>Results – Prognostic value in ARIC study participants with preserved LVEF<br>Results – Performance in detecting elevated LVFP<br>Tables 3, 4, and 5, Figures 3 and 5<br>Supplemental Tables 6, 7, and 8<br>Supplemental Figures 1, 2, 3, 4, and 5<br>Also reported previously in the paper describing the development and initial validation of the DL model: https://doi.org/10.1016/j.jcmg.2021.04.010
7.2 Discussing study strengths	
Articulate strengths	Discussion
7.3 Reporting on bias and fairness assessment	
Report stratified performance metrics for demographic subgroups	Results – Prognostic value in the entire ARIC cohort<br>Results – Prognostic value in ARIC study participants with preserved LVEF<br>Results – Performance in detecting elevated LVFP<br>Tables 3, 4, and 5, Figures 3 and 5<br>Supplemental Tables 6, 7, and 8<br>Supplemental Figures 1, 2, 3, 4, and 5
Describe bias mitigation strategies and residual bias	Discussion – Limitations
Acknowledge fairness evaluation limitations	Discussion – Limitations
7.4 Contextualizing with alternative methods	
Benchmark against clinical standards/traditional risk scores	The objective of this study was to compare the diagnostic and prognostic performance of the DL model with the 2016 ASE/EACVI and 2025 ASE guidelines.
Justify complex AI models over simpler alternatives	Justified previously in the paper reporting the development and initial validation of the DL model: https://doi.org/10.1016/j.jcmg.2021.04.010
Discuss complementary methods for validation	N/A

AI – artificial intelligence, ASE – American Society of Echocardiography, DL – deep learning, EACVI – European Association of Cardiovascular Imaging, LVFP – left ventricular filling pressure; other abbreviations as in Supplemental Table 1.

Supplemental Table 3. Clinical and echocardiographic characteristics of patients reaching and not reaching the composite endpoint of HF hospitalization or all-cause death in the ARIC cohort

	**Missing** n (%)	**All participants** n=5,450	**Participants reaching the composite endpoint** n=1,320	**Participant not reaching the composite endpoint** n=4,130	**P-value**
**Demographics, vitals, risk factors**					
Age, years	0 (0)	75 (71–79)	78 (73–83)	74 (71–78)	<0.001
Male sex	0 (0)	2,326 (43)	681 (52)	1,645 (40)	<0.001
Black race	0 (0)	1,049 (19)	236 (18)	813 (20)	0.159
BMI, kg/m^2	31 (1)	27.9 (24.9–31.5)	27.9 (24.7–31.9)	27.9 (24.9–31.4)	0.851
BSA, m^2	7 (0)	1.86 (1.70–2.01)	1.88 (1.70–2.03)	1.85 (1.70–2.01)	0.093
SBP, mmHg	14 (0)	129 (118–141)	131 (119–143)	128 (118–140)	<0.001
DBP, mmHg	14 (0)	66 (59–74)	64 (57–72)	67 (60–74)	<0.001
HR, 1/min	14 (0)	64 (57–71)	65 (58–73)	64 (57–71)	<0.001
Hypertension	0 (0)	4,391 (81)	1,165 (88)	3,226 (78)	<0.001
Diabetes	0 (0)	2,004 (37)	626 (47)	1,378 (33)	<0.001
Atrial fibrillation	0 (0)	166 (3)	84 (6)	82 (2)	<0.001
Chronic kidney disease	0 (0)	1,500 (28)	522 (40)	978 (24)	<0.001
History of HF	0 (0)	908 (17)	434 (33)	474 (11)	<0.001
History of CHD	0 (0)	1,048 (19)	425 (32)	623 (15)	<0.001
History of non-cardiac PH	0 (0)	120 (2)	73 (6)	47 (1)	<0.001
History of stroke	0 (0)	208 (4)	91 (7)	117 (3)	<0.001
**Laboratory results**					
Hemoglobin, g/dL	152 (3)	13.3 (12.4–14.2)	13.3 (12.4–14.2)	13.3 (12.4–14.2)	0.777
Creatinine, mg/dL	25 (0)	0.92 (0.78–1.10)	0.99 (0.84–1.24)	0.90 (0.77–1.06)	<0.001
GFR, mL/min/1.73m^2	25 (0)	71 (59–83)	65 (52–79)	72 (61–83)	<0.001
NT-proBNP, pg/mL	163 (3)	134 (69–264)	247 (122–590)	111 (60–210)	<0.001
**Echocardiographic parameters**					
IVSd, mm	36 (1)	10 (9–11)	11 (10–12)	1 (9–11)	<0.001
LVPWd, mm	27 (0)	9 (8–10)	9 (9–11)	9 (8–10)	<0.001
LVIDd, mm	37 (1)	44 (41–47)	45 (41–49)	43 (40–47)	<0.001
LVIDs, mm	32 (1)	26 (23–29)	27 (24–32)	26 (23–29)	<0.001
LVMi, g/m^2	46 (1)	76.4 (66.1–89.1)	83.6 (71.8–102.4)	74.3 (65.0–86.2)	<0.001
LVEDVi, mL/m^2	166 (3)	42.4 (36.4–49.7)	44.5 (37.9–53.4)	41.8 (36.0–48.8)	<0.001
LVESVi, mL/m^2	166 (3)	14.4 (11.6–17.9)	15.6 (12.4–20.5)	14.1 (11.5–17.2)	<0.001
LVEF, %	160 (3)	65.8 (61.9–69.2)	64.3 (59.4–68.2)	66 (62.5–69.5)	<0.001
LAVi, mL/m^2	54 (1)	24.7 (20.3–30.2)	27.8 (22.4–34.6)	24.0 (19.7–28.9)	<0.001
Moderate or greater AS	0 (0)	40 (1)	24 (2)	16 (0)	<0.001
Moderate or greater AR	0 (0)	22 (0)	9 (1)	13 (0)	0.114
Moderate MR	0 (0)	98 (2)	43 (3)	55 (1)	<0.001
E, cm/s	0 (0)	65 (54–78)	68 (55–84)	64 (54–76)	<0.001
E-DT, ms	19 (0)	200 (173–230)	197 (170–229)	203 (177–230)	<0.001
A, cm/s	229 (4)	79 (67–91)	82 (69–96)	78 (67–90)	<0.001
E/A	229 (4)	0.8 (0.7–1.0)	0.8 (0.6–1.0)	0.8 (0.7–1.0)	0.050

Supplemental Table 3. Continued

	**Missing** n (%)	**All participants** n=5,450	**Participants reaching the composite endpoint** n=1,320	**Participant not reaching the composite endpoint** n=4,130	**P-value**
e' (septal), cm/s	0 (0)	5.5 (4.7–6.5)	5.1 (4.4–6.1)	5.6 (4.8–6.6)	<0.001
E/e' (septal)	0 (0)	11.6 (9.5–14.4)	12.9 (10.2–16.5)	11.3 (9.3–13.8)	<0.001
e' (lateral), cm/s	15 (0)	6.8 (5.6–8.2)	6.5 (5.2–8.0)	6.9 (5.7–8.3)	<0.001
E/e' (lateral)	15 (0)	9.5 (7.6–12.0)	10.3 (8.0–13.3)	9.3 (7.5–11.6)	<0.001
e' (average), cm/s	15 (0)	6.2 (5.3–7.3)	5.9 (4.9–7.0)	6.3 (5.4–7.4)	<0.001
E/e' (average)	15 (0)	10.6 (8.7–13.1)	11.8 (9.4–14.8)	10.3 (8.5–12.6)	<0.001
TRV, m/s	2,273 (42)	2.36 (2.18–2.55)	2.45 (2.24–2.68)	2.34 (2.17–2.52)	<0.001
2016 ASE/EACVI guidelines	0 (0)				
Normal diastolic function		1,432 (26)	172 (12)	1,260 (31)	
Indet. diastolic function		307 (6)	56 (4)	251 (6)	
DD with normal LAP		2,578 (47)	621 (47)	1,957 (47)	
DD with indeterminate LAP		775 (14)	294 (22)	481 (12)	
DD with elevated LAP		358 (7)	177 (13)	181 (4)	<0.001
2025 ASE guidelines	0 (0)				
Normal diastolic function		1,246 (23)	191 (14)	1,055 (26)	
DD with normal LAP		3,585 (66)	822 (62)	2,763 (67)	
DD with indeterminate LAP		124 (2)	69 (5)	55 (1)	
DD with elevated LAP		495 (9)	238 (18)	257 (6)	<0.001
DL-predicted probability of DD	0 (0)	0.30 (0.04–0.86)	0.80 (0.14–0.97)	0.18 (0.04–0.74)	<0.001

Continuous variables are presented as median (interquartile range) and categorical variables as n (%). Comparisons between patients reaching and not reaching the composite endpoint were performed using unpaired Student's t-test or Mann-Whitney U test for continuous variables, Chi-squared or Fisher's exact test for categorical variables, as appropriate. All characteristics shown in this table were collected during Visit 5.

AR – aortic regurgitation, AS – aortic stenosis, BMI – body mass index, BSA – body surface area, CHD – coronary heart disease, DBP – diastolic blood pressure, DD – diastolic dysfunction, GFR – glomerular filtration rate, HF – heart failure, HR – heart rate, IVSd – thickness of the interventricular septum at end-diastole, LAP – left atrial pressure, LVEDVi – left ventricular end-diastolic volume index, LVESVi – left ventricular end-systolic volume index, LVIDd – left ventricular internal diameter at end-diastole, LVIDs – left ventricular internal diameter at end-systole, LVPWd – thickness of the left ventricular posterior wall at end-diastole, MR – mitral regurgitation, NT-proBNP – N-terminal pro-brain natriuretic peptide, PH – pulmonary hypertension, SBP – systolic blood pressure; other abbreviations as in Supplemental Tables 1 and 2.

Supplemental Table 4. Univariable Cox regression models predicting the composite endpoint of HF hospitalization or all-cause death in the ARIC cohort

	N	HR (95% CI)	P-value
**Demographics and risk factors**			
Age	5,450	1.106 (1.095–1.117)	<0.001
Male sex	5,450	1.505 (1.351–1.676)	<0.001
Black race	5,450	1.081 (0.938–1.246)	0.283
Hypertension	5,450	2.006 (1.696–2.372)	<0.001
Diabetes	5,450	1.710 (1.534–1.905)	<0.001
Atrial fibrillation	5,450	2.769 (2.220–3.454)	<0.001
Chronic kidney disease	5,450	1.902 (1.703–2.124)	<0.001
History of HF	5,450	3.267 (2.912–3.666)	<0.001
History of CHD	5,450	2.340 (2.085–2.627)	<0.001
History of non-cardiac PH	5,450	3.761 (2.970–4.763)	<0.001
History of stroke	5,450	2.203 (1.780–2.726)	<0.001
**Laboratory results**			
Hemoglobin	5,298	0.991 (0.955–1.028)	0.615
Creatinine	5,425	1.689 (1.587–1.797)	<0.001
GFR	5,425	0.982 (0.979–0.985)	<0.001
Log_{10}(NT-proBNP)	5,287	5.410 (4.820–6.071)	<0.001
**Echocardiographic parameters**			
LVMi	5,404	1.024 (1.022–1.026)	<0.001
LVEDVi	5,284	1.030 (1.026–1.035)	<0.001
LVESVi	5,284	1.057 (1.052–1.063)	<0.001
LVEF	5,290	0.940 (0.934–0.947)	<0.001
LAVi	5,396	1.027 (1.025–1.030)	<0.001
Moderate or greater AS	5,450	3.631 (2.425–5.438)	<0.001
Moderate or greater AR	5,450	2.060 (1.069–3.968)	0.031
Moderate MR	5,450	2.186 (1.613–2.963)	<0.001
E	5,450	1.013 (1.010–1.016)	<0.001
E-DT	5,431	0.997 (0.996–0.999)	<0.001
A	5,221	1.009 (1.007–1.012)	<0.001
E/A	5,221	1.267 (1.042–1.540)	0.018
e' (septal)	5,450	0.819 (0.785–0.853)	<0.001
E/e' (septal)	5,450	1.087 (1.077–1.096)	<0.001
e' (lateral)	5,435	0.934 (0.909–0.961)	<0.001
E/e' (lateral)	5,435	1.079 (1.067–1.092)	<0.001
e' (average)	5,435	0.867 (0.835–0.900)	<0.001
E/e' (average)	5,435	1.095 (1.084–1.107)	<0.001
TRV	3,177	3.630 (2.923–4.507)	<0.001
2016 ASE/EACVI guidelines	5,450		
Indeterminate diastolic function		1.549 (1.146–2.094)	0.004
DD with normal LAP		2.212 (1.868–2.620)	<0.001
DD with indeterminate LAP		3.905 (3.235–4.714)	<0.001
DD with elevated LAP		5.624 (4.558–6.938)	<0.001

Supplemental Table 4. Continued

	N	HR (95% CI)	P-value
2025 ASE guidelines	5,450		
DD with normal LAP		1.593 (1.361–1.865)	<0.001
DD with indeterminate LAP		5.067 (3.847–6.674)	<0.001
DD with elevated LAP		4.141 (3.423–5.010)	<0.001
DL-predicted probability of DD	5,450	4.632 (4.000–5.364)	<0.001
DL-predicted probability of DD ≥0.50	5,450	2.744 (2.455–3.067)	<0.001

Abbreviations as in Supplemental Tables 1, 2, and 3.

Supplemental Table 5. Univariable and multivariable Cox regression models predicting the composite endpoint of HF hospitalization or all-cause death in the ARIC cohort (indeterminate groups excluded)

	**Univariable models**		**Multivariable model #1** C-index: 0.731 (0.715–0.747)		**Multivariable model #2** C-index: 0.723 (0.709–0.737)		**Multivariable model #3** C-index: 0.739 (0.725–0.753)	
	HR (95% CI)	P-value	HR (95% CI)	P-value	HR (95% CI)	P-value	HR (95% CI)	P-value
Age	1.106 (1.095–1.117)	<0.001	1.087 (1.074–1.101)	<0.001	1.084 (1.072–1.096)	<0.001	1.075 (1.064–1.087)	<0.001
Male sex	1.505 (1.351–1.676)	<0.001	1.374 (1.207–1.565)	<0.001	1.340 (1.195–1.503)	<0.001	1.286 (1.150–1.438)	<0.001
Hypertension	2.006 (1.696–2.372)	<0.001	1.176 (0.964–1.435)	0.110	1.144 (0.958–1.366)	0.136	1.078 (0.905–1.285)	0.400
Diabetes	1.710 (1.534–1.905)	<0.001	1.365 (1.198–1.555)	<0.001	1.460 (1.302–1.637)	<0.001	1.375 (1.230–1.537)	<0.001
Atrial fibrillation	2.769 (2.220–3.454)	<0.001	1.406 (0.349–5.671)	0.632	1.671 (1.262–2.213)	<0.001	1.554 (1.240–1.949)	<0.001
Chronic kidney disease	1.902 (1.703–2.124)	<0.001	1.395 (1.220–1.594)	<0.001	1.386 (1.232–1.558)	<0.001	1.404 (1.253–1.574)	<0.001
History of HF	3.267 (2.912–3.666)	<0.001	1.918 (1.647–2.233)	<0.001	2.106 (1.843–2.407)	<0.001	2.037 (1.791–2.317)	<0.001
History of CHD	2.340 (2.085–2.627)	<0.001	1.132 (0.971–1.319)	0.114	1.267 (1.107–1.451)	<0.001	1.206 (1.058–1.376)	0.005
History of non-cardiac PH	3.761 (2.970–4.763)	<0.001	1.836 (1.357–2.485)	<0.001	1.155 (0.816–1.634)	0.416	1.690 (1.324–2.157)	<0.001
History of stroke	2.203 (1.780–2.726)	<0.001	1.110 (0.856–1.439)	0.433	1.143 (0.911–1.433)	0.248	1.062 (0.853–1.321)	0.591
2016 ASE/EACVI guidelines								
DD with normal LAP	2.215 (1.871–2.623)	<0.001	1.438 (1.198–1.727)	<0.001				
DD with elevated LAP	5.659 (4.587–6.982)	<0.001	2.791 (2.217–3.513)	<0.001				
2025 ASE guidelines								
DD with normal LAP	1.594 (1.362–1.866)	<0.001			1.232 (1.050–1.446)	0.011		
DD with elevated LAP	4.147 (3.427–5.017)	<0.001			2.216 (1.812–2.711)	<0.001		
DL-predicted probability of DD	4.632 (4.000–5.364)	<0.001					2.673 (2.293–3.117)	<0.001

Only clinically relevant variables without missing values were considered for the multivariable models. The two guidelines classified different patients as indeterminate; therefore, their associations with outcomes were evaluated in overlapping but non-identical subsets after excluding indeterminate cases: the 2016 ASE/EACVI guidelines were evaluated in 4,368 of 5,450 (80%) patients, whereas the 2025 ASE guidelines were evaluated in 5,326 of 5,450 (98%) patients. The DL model was evaluated in all patients (n=5,450). Abbreviations as in Supplemental Tables 1, 2, and 3.

Supplemental Table 6. Incremental prognostic value of the guideline-based classifications and the DL model over NT-proBNP in the ARIC cohort

	C-index	ΔC-index	P-value
**NT-proBNP**	0.703 (0.688–0.718)		
NT-proBNP + **2016 ASE/EACVI guidelines**	0.715 (0.700–0.730)	+0.012	<0.001
NT-proBNP + **2025 ASE guidelines**	0.710 (0.695–0.725)	+0.008	<0.001
NT-proBNP + **DL-predicted probability of DD**	0.720 (0.705–0.735)	+0.017	<0.001

Each row corresponds to a Cox regression model predicting the composite endpoint of HF hospitalization or all-cause death, with the variable(s) in the first column included as predictors. The third column shows the difference in the C-index of the given model vs. that of the model containing NT-proBNP only, whereas the P-value in the fourth column refers to the significance of this difference. Abbreviations as in Supplemental Tables 1, 2, and 3.

Supplemental Table 7. Prognostic performance of the guidelines and the DL model in the ARIC cohort using time-dependent receiver operating characteristic curves

	1st year	2nd year	3rd year	4th year	5th year	6th year	7th year
**2016 ASE/EACVI guidelines**	0.716 (0.677–0.756)	0.680 (0.652–0.708)	0.680 (0.657–0.702)	0.668 (0.649–0.687)	0.658 (0.641–0.675)	0.658 (0.641–0.674)	0.655 (0.634–0.677)
**2025 ASE guidelines**	0.667 (0.625–0.710)	0.645 (0.617–0.674)	0.637 (0.614–0.659)	0.627 (0.608–0.646)	0.614 (0.597–0.631)	0.607 (0.591–0.623)	0.625 (0.606–0.645)
**DL model**	0.787 (0.745–0.829)	0.741 (0.711–0.771)	0.729 (0.704–0.753)	0.719 (0.698–0.740)	0.702 (0.682–0.721)	0.691 (0.673–0.709)	0.710 (0.690–0.731)
**DL vs. 2016 guidelines**	p<0.001	p<0.001	p<0.001	p<0.001	p<0.001	p<0.001	p<0.001
**DL vs. 2025 guidelines**	p<0.001	p<0.001	p<0.001	p<0.001	p<0.001	p<0.001	p<0.001
**2025 vs. 2016 guidelines**	p=0.007	p=0.011	p<0.001	p<0.001	p<0.001	p<0.001	p=0.007

The composite of HF hospitalization or all-cause death was used as the endpoint in this analysis. Areas under the receiver operating characteristic curve were calculated at the end of each year up to 7 years of follow-up. Abbreviations as in Supplemental Tables 1 and 2.

Supplemental Table 8. C-indices of univariable Cox regression models predicting the composite endpoint of HF hospitalization or all-cause death across different subgroups of the ARIC cohort

	2016 guidelines	2025 guidelines	DL model	DL vs. 2016 guidelines	DL vs. 2025 guidelines	2025 vs. 2016 guidelines
**All participants**	0.638 (0.624–0.652)	0.602 (0.588–0.616)	0.676 (0.660–0.692)	p<0.001	p<0.001	p<0.001
**Race**						
Black (n=1,049)	0.617 (0.584–0.650)	0.596 (0.564–0.628)	0.677 (0.640–0.714)	p<0.001	p<0.001	p=0.196
White (n=4,401)	0.643 (0.627–0.659)	0.603 (0.588–0.618)	0.676 (0.659–0.693)	p<0.001	p<0.001	p<0.001
**Atrial fibrillation**						
Yes (n=166)	0.510 (0.495–0.525)	0.556 (0.498–0.614)	0.665 (0.604–0.726)	p<0.001	p=0.001	p=0.119
No (n=5,284)	0.630 (0.615–0.645)	0.594 (0.580–0.608)	0.672 (0.656–0.688)	p<0.001	p<0.001	p<0.001
**History of HF**						
Yes (n=908)	0.600 (0.576–0.624)	0.612 (0.588–0.636)	0.689 (0.664–0.714)	p<0.001	p<0.001	p=0.292
No (n=4,542)	0.607 (0.589–0.625)	0.573 (0.557–0.589)	0.636 (0.616–0.656)	p=0.004	p<0.001	p<0.001
**History of CHD**						
Yes (n=1,048)	0.605 (0.581–0.629)	0.612 (0.588–0.636)	0.679 (0.653–0.705)	p<0.001	p<0.001	p=0.564
No (n=4,402)	0.621 (0.603–0.639)	0.586 (0.570–0.602)	0.655 (0.636–0.674)	p=0.004	p<0.001	p<0.001
**Elevated NT-proBNP**						
Yes (n=1,531)	0.659 (0.638–0.680)	0.622 (0.601–0.643)	0.697 (0.675–0.719)	p<0.001	p<0.001	p<0.001
No (n=3,756)	0.580 (0.561–0.599)	0.561 (0.543–0.579)	0.622 (0.600–0.644)	p<0.001	p<0.001	p=0.078
**Availability of TRV**						
Yes (n=3,177)	0.650 (0.632–0.668)	0.617 (0.599–0.635)	0.687 (0.667–0.707)	p<0.001	p<0.001	p<0.001
No (n=2,273)	0.630 (0.607–0.653)	0.577 (0.556–0.598)	0.658 (0.633–0.683)	p=0.019	p<0.001	p<0.001

NT-proBNP levels were considered elevated at ≥125 pg/mL for patients younger than 75 years and ≥450 pg/mL for those aged 75 years or older.

Abbreviations as in Supplemental Tables 1, 2, and 3.

Supplemental Table 9. Pairwise comparisons of the event-free survival of patients classified according to the 2016 ASE/EACVI guidelines

	Indeterminate diastolic function	DD with normal LAP	DD with indet. LAP	DD with elevated LAP
**Normal diastolic function**	p=0.004	p<0.001	p<0.001	p<0.001
**Indeterminate diastolic function**		p=0.009	p<0.001	p<0.001
**DD with normal LAP**			p<0.001	p<0.001
**DD with indet. LAP**				p<0.001

The composite of HF hospitalization or all-cause death was used as the endpoint in this analysis. Cell values are adjusted P-values of pairwise Log-rank tests. P-values were adjusted using the Benjamini-Hochberg method. Abbreviations as in Supplemental Tables 2 and 3.

Supplemental Table 10. Pairwise comparisons of the event-free survival of patients classified according to the 2025 ASE guidelines

	DD with normal LAP	DD with indeterminate LAP	DD with elevated LAP
**Normal diastolic function**	p<0.001	p<0.001	p<0.001
**DD with normal LAP**		p<0.001	p<0.001
**DD with indeterminate LAP**			p=0.150

The composite of HF hospitalization or all-cause death was used as the endpoint in this analysis. Cell values are adjusted P-values of pairwise Log-rank tests. P-values were adjusted using the Benjamini-Hochberg method. Abbreviations as in Supplemental Tables 2 and 3.

Supplemental Table 11. Univariable and multivariable Fine-Gray competing risk models predicting HF hospitalization in the ARIC cohort

	Univariable models		Multivariable model #1 C-index: 0.770 (0.753–0.787) AIC: 11,228		Multivariable model #2 C-index: 0.765 (0.747–0.783) AIC: 11,238		Multivariable model #3 C-index: 0.788 (0.771–0.805) AIC: 11,121	
	sHR (95% CI)	P-value	sHR (95% CI)	P-value	sHR (95% CI)	P-value	sHR (95% CI)	P-value
Age	1.079 (1.064–1.093)	<0.001	1.039 (1.024–1.054)	<0.001	1.038 (1.023–1.053)	<0.001	1.031 (1.016–1.046)	<0.001
Male sex	1.544 (1.333–1.789)	<0.001	1.349 (1.155–1.576)	<0.001	1.302 (1.116–1.517)	<0.001	1.211 (1.039–1.413)	0.015
Hypertension	3.075 (2.339–4.042)	<0.001	1.538 (1.160–2.039)	0.003	1.595 (1.202–2.116)	0.001	1.424 (1.070–1.894)	0.015
Diabetes	1.963 (1.695–2.273)	<0.001	1.431 (1.234–1.660)	<0.001	1.424 (1.227–1.653)	<0.001	1.341 (1.155–1.557)	<0.001
Atrial fibrillation	2.071 (2.021–3.608)	<0.001	1.040 (0.741–1.458)	0.822	1.168 (0.847–1.611)	0.344	1.170 (0.865–1.583)	0.309
Chronic kidney disease	2.052 (1.769–2.380)	<0.001	1.381 (1.184–1.612)	<0.001	1.428 (1.224–1.666)	<0.001	1.424 (1.221–1.660)	<0.001
History of HF	4.699 (4.055–5.445)	<0.001	2.550 (2.161–3.009)	<0.001	2.721 (2.308–3.208)	<0.001	2.503 (2.125–2.949)	<0.001
History of CHD	3.005 (2.585–3.494)	<0.001	1.362 (1.147–1.618)	<0.001	1.453 (1.226–1.722)	<0.001	1.323 (1.116–1.569)	0.001
History of non-cardiac PH	5.234 (3.973–6.895)	<0.001	2.023 (1.496–2.736)	<0.001	1.520 (1.097–2.106)	0.012	1.895 (1.404–2.557)	<0.001
History of stroke	2.424 (1.852–3.174)	<0.001	1.077 (0.799–1.451)	0.625	1.036 (0.766–1.402)	0.818	1.003 (0.749–1.345)	0.982
2016 ASE/EACVI guidelines								
Indeterminate diastolic function	1.908 (1.218–2.989)	0.005	1.749 (1.112–2.750)	0.015				
DD with normal LAP	2.858 (2.187–3.733)	<0.001	1.554 (1.170–2.063)	0.002				
DD with indeterminate LAP	5.875 (4.420–7.808)	<0.001	2.588 (1.886–3.550)	<0.001				
DD with elevated LAP	10.136 (7.531–13.643)	<0.001	4.213 (3.034–5.850)	<0.001				
2025 ASE guidelines								
DD with normal LAP	1.963 (1.539–2.504)	<0.001			1.585 (1.239–2.028)	<0.001		
DD with indeterminate LAP	7.109 (4.886–10.342)	<0.001			2.267 (1.462–3.517)	<0.001		
DD with elevated LAP	7.089 (5.424–9.264)	<0.001			3.669 (2.761–4.875)	<0.001		
DL-predicted probability of DD	9.276 (7.314–11.760)	<0.001					4.931 (3.862–6.296)	<0.001

The AIC values of the univariable models containing the 2016 ASE/EACVI guideline classifications, the 2025 ASE guideline classifications, and the DL-predicted probability of DD were 11,598, 11,638, and 11,449, respectively. Only clinically relevant variables without missing values were considered for the multivariable models. AIC – Akaike information criterion, sHR – subdistribution hazard ratio; other abbreviations as in Supplemental Tables 1, 2, and 3.

Supplemental Table 12. Univariable and multivariable Cox regression models predicting all-cause death in the ARIC cohort

	Univariable models		Multivariable model #1<br>C-index: 0.720 (0.703–0.737)<br>AIC: 15,296		Multivariable model #2<br>C-index: 0.717 (0.700–0.734)<br>AIC: 15,310		Multivariable model #3<br>C-index: 0.722 (0.705–0.739)<br>AIC: 15,281	
	HR (95% CI)	P-value	HR (95% CI)	P-value	HR (95% CI)	P-value	HR (95% CI)	P-value
Age	1.119 (1.106–1.132)	<0.001	1.097 (1.084–1.111)	<0.001	1.098 (1.085–1.112)	<0.001	1.094 (1.081–1.108)	<0.001
Male sex	1.544 (1.359–1.754)	<0.001	1.402 (1.229–1.600)	<0.001	1.389 (1.217–1.584)	<0.001	1.351 (1.184–1.541)	<0.001
Hypertension	1.728 (1.430–2.088)	<0.001	1.023 (0.839–1.248)	0.819	1.057 (0.867–1.289)	0.582	1.014 (0.831–1.236)	0.894
Diabetes	1.600 (1.409–1.819)	<0.001	1.390 (1.218–1.585)	<0.001	1.396 (1.224–1.593)	<0.001	1.369 (1.200–1.563)	<0.001
Atrial fibrillation	2.981 (2.333–3.809)	<0.001	1.525 (1.143–2.033)	0.004	1.508 (1.140–1.994)	0.004	1.714 (1.333–2.205)	<0.001
Chronic kidney disease	1.903 (1.671–2.167)	<0.001	1.392 (1.217–1.592)	<0.001	1.419 (1.241–1.623)	<0.001	1.408 (1.231–1.610)	<0.001
History of HF	2.612 (2.274–3.000)	<0.001	1.656 (1.414–1.939)	<0.001	1.717 (1.467–2.009)	<0.001	1.676 (1.432–1.961)	<0.001
History of CHD	1.993 (1.736–2.289)	<0.001	1.088 (0.928–1.274)	0.299	1.151 (0.984–1.346)	0.078	1.101 (0.941–1.288)	0.230
History of non-cardiac PH	3.331 (2.537–4.374)	<0.001	1.642 (1.238–2.179)	<0.001	1.275 (0.933–1.742)	0.127	1.575 (1.187–2.090)	0.002
History of stroke	1.938 (1.495–2.512)	<0.001	1.112 (0.853–1.448)	0.433	1.103 (0.847–1.438)	0.467	1.064 (0.817–1.387)	0.644
2016 ASE/EACVI guidelines								
Indeterminate diastolic function	1.469 (1.039–2.078)	0.030	1.245 (0.879–1.765)	0.218				
DD with normal LAP	1.951 (1.608–2.368)	<0.001	1.335 (1.087–1.640)	0.006				
DD with indeterminate LAP	3.350 (2.698–4.159)	<0.001	1.715 (1.340–2.195)	<0.001				
DD with elevated LAP	4.282 (3.349–5.476)	<0.001	2.188 (1.681–2.848)	<0.001				
2025 ASE guidelines								
DD with normal LAP	1.424 (1.189–1.706)	<0.001			1.093 (0.910–1.314)	0.340		
DD with indeterminate LAP	5.329 (3.954–7.182)	<0.001			1.874 (1.296–2.711)	<0.001		
DD with elevated LAP	3.067 (2.450–3.839)	<0.001			1.598 (1.260–2.026)	<0.001		
DL-predicted probability of DD	3.346 (2.827–3.960)	<0.001					1.883 (1.575–2.251)	<0.001

The AIC values of the univariable models containing the 2016 ASE/EACVI guideline classifications, the 2025 ASE guideline classifications, and the DL-predicted probability of DD were 15,723, 15,750, and 15,703, respectively. Only clinically relevant variables without missing values were considered for the multivariable models. Abbreviations as in Supplemental Tables 1, 2, 3, and 11.

Supplemental Table 13. Clinical and echocardiographic characteristics of patients with elevated and normal invasively measured LVFP in the first hemodynamic validation cohort

	Missing n (%)	All patients n=83	Elevated LVFP n=25	Normal LVFP n=58	P-value
**Demographics, vitals, risk factors**					
Age, years	0 (0)	61 (54–66)	58 (53–64)	63 (54–67)	0.306
Male sex	0 (0)	56 (67)	19 (76)	37 (64)	0.404
Black race	0 (0)	3 (4)	2 (8)	1 (2)	0.214
BMI, kg/m^2	0 (0)	30.8 (27.6–34.7)	33.1 (30.8–37.1)	29.1 (27.0–34.0)	0.006
BSA, m^2	0 (0)	2.11 (1.94–2.28)	2.2 (2.09–2.34)	2.08 (1.92–2.22)	0.014
SBP, mmHg	0 (0)	133 (117–147)	131 (112–148)	133 (119–146)	0.623
DBP, mmHg	0 (0)	77 (69–89)	79 (68–87)	76.5 (69–90)	0.732
HR, 1/min	1 (1)	73 (65–85)	83 (73–93)	68 (62–81)	<0.001
Hypertension	0 (0)	82 (99)	24 (96)	58 (100)	0.301
Diabetes	0 (0)	42 (51)	16 (64)	26 (45)	0.173
Atrial fibrillation	0 (0)	6 (7)	6 (24)	0 (0)	<0.001
Chronic kidney disease	0 (0)	14 (17)	7 (28)	7 (12)	0.145
History of HF	0 (0)	30 (36)	19 (76)	11 (19)	<0.001
History of CHD	0 (0)	67 (81)	15 (60)	52 (90)	0.005
History of stroke	0 (0)	10 (12)	3 (12)	7 (12)	1.000
**Laboratory results**					
Hemoglobin, g/dL	59 (71)	11.5 (9.6–12.3)	11.7 (9.3–12.4)	10.6 (10.0–11.6)	0.446
Creatinine, mg/dL	2 (2)	1.00 (0.83–1.38)	1.32 (0.88–1.71)	0.98 (0.82–1.17)	0.005
GFR, mL/min/1.73m^2	2 (2)	68 (47–87)	53 (34–86)	73 (54–88)	0.031
BNP, pg/mL	56 (67)	1,068 (425–2,170)	1,262 (425–2,206)	1,027 (462–1,862)	0.700
**Echocardiographic parameters**					
IVSd, mm	2 (2)	11 (11–14)	11 (10–12)	12 (11–15)	0.037
LVPWd, mm	2 (2)	10 (9–12)	11 (9–12)	9 (8–11)	0.045
LVIDd, mm	26 (31)	46 (42–50)	52 (47–56)	45 (42–50)	0.012
LVIDs, mm	26 (31)	33 (29–35)	34 (33–42)	32 (29–34)	0.074
LVMi, g/m^2	5 (6)	99.0 (80.8–118.3)	116.9 (103.5–141.9)	90.1 (73.7–107.3)	<0.001
LVEDVi, mL/m^2	2 (2)	57.3 (47.7–76.0)	81.2 (62.7–97.3)	53.1 (44.2–63.9)	<0.001
LVESVi, mL/m^2	3 (4)	26.9 (19.6–40.3)	47.1 (31.2–62.6)	22.7 (18.6–29.8)	<0.001
LVEF, %	0 (0)	54.0 (38.0–59.5)	32.0 (17.0–47.0)	56 (51.5–61.8)	<0.001
LAVi, mL/m^2	3 (4)	27.9 (20.7–38.7)	40.7 (35.1–52.9)	24.55 (20.0–29.6)	<0.001
LARS, %	18 (22)	21.4 (11.0–30.5)	10.7 (7.8–15.0)	27.0 (18.4–34.1)	<0.001
Moderate or greater AS	0 (0)	3 (4)	1 (4)	2 (3)	1.000
Moderate or greater AR	0 (0)	1 (1)	0 (0)	1 (2)	1.000
Moderate MR	0 (0)	11 (13)	9 (36)	2 (3)	<0.001
E, cm/s	0 (0)	81 (63–95)	101 (87–115)	72 (60–86)	<0.001
E-DT, ms	1 (1)	202 (192–232)	193 (160–208)	210 (194–235)	0.009
A, cm/s	5 (6)	70 (56–85)	67 (41–84)	70 (59–85)	0.250
E/A	5 (6)	1.0 (0.8–1.5)	1.2 (1.0–2.8)	1.0 (0.8–1.2)	0.005
IVRT, ms	21 (25)	77 (70–90)	76 (69–80)	79 (72–90)	0.150

Supplemental Table 13. Continued

	Missing n (%)	All patients n=83	Elevated LVFP n=25	Normal LVFP n=58	P-value
e' (septal), cm/s	0 (0)	6.0 (4.7–7.3)	5 (3.3–6.0)	6.2 (5.0–8.0)	<0.001
E/e' (septal)	0 (0)	12.7 (9.5–20.0)	21.3 (17.0–28.6)	11.0 (9.1–13.5)	<0.001
e' (lateral), cm/s	2 (2)	8.0 (6.0–9.9)	6 (5.1–9.6)	8.8 (7.1–9.9)	0.012
E/e' (lateral)	2 (2)	9.1 (6.9–14.6)	17.3 (12.8–21.7)	7.9 (6.5–11.5)	<0.001
e' (average), cm/s	2 (2)	7.5 (5.5–8.4)	5.5 (4.7–7.3)	7.6 (6.2–8.7)	0.001
E/e' (average)	2 (2)	10.1 (8.0–17.8)	17.9 (15.3–22.8)	9.5 (7.7–11.9)	<0.001
TRV, m/s	39 (47)	2.54 (1.87–2.95)	2.64 (2.23–3.00)	2.29 (1.57–2.77)	0.139
PASP, mmHg	52 (63)	24 (13–37)	37 (15–47)	21 (12–31)	0.189
2016 ASE/EACVI guidelines	0 (0)				
Normal diastolic function		1 (1)	0 (0)	1 (2)	
Indeterminate diastolic function		0 (0)	0 (0)	0 (0)	
DD with normal LAP		44 (53)	4 (16)	40 (69)	
DD with indeterminate LAP		16 (19)	6 (24)	10 (17)	
DD with elevated LAP		22 (27)	15 (60)	7 (12)	<0.001
2025 ASE guidelines	0 (0)				
Normal diastolic function		34 (41)	3 (12)	31 (53)	
DD with normal LAP		25 (30)	5 (20)	20 (34)	
DD with indeterminate LAP		4 (5)	3 (12)	1 (2)	
DD with elevated LAP		20 (24)	14 (56)	6 (10)	<0.001
DL-predicted probability of DD	0 (0)	0.95 (0.21–1.00)	1.00 (1.00–1.00)	0.80 (0.07–0.99)	<0.001
**Invasive hemodynamic assessment**					
LVFP, mmHg	0 (0)	11.1 (7.7–17.5)	24.0 (18.1–27.0)	8.7 (5.6–11.1)	<0.001

LVFP was measured as PCWP by right heart catheterization or LV pre-atrial contraction pressure by left heart catheterization. Continuous variables are presented as median (interquartile range) and categorical variables as n (%). Comparisons between subgroups with elevated and normal LVFP were performed using unpaired Student's t-test or Mann-Whitney U test for continuous variables, Chi-squared or Fisher's exact test for categorical variables, as appropriate.

PCWP – pulmonary capillary wedge pressure, LV – left ventricular; other abbreviations as in Supplemental Tables 1, 2, and 3.

Supplemental Table 14. Clinical and echocardiographic characteristics of patients with elevated and normal invasively measured LVFP in the second hemodynamic validation cohort

	**Missing** n (%)	**All patients** n=130	**Elevated LVFP** n=30	**Normal LVFP** n=100	**P-value**
**Demographics, vitals, risk factors**					
Age, years	0 (0)	66 (51–75)	67 (61–72)	65 (48–75)	0.241
Male sex	0 (0)	70 (54)	24 (80)	46 (46)	0.002
BMI, kg/m^2	3 (2)	22.0 (19.4–24.7)	22.2 (20.1–24.1)	21.5 (19.3–24.8)	0.583
BSA, m^2	3 (2)	1.58 (1.43–1.73)	1.65 (1.53–1.76)	1.55 (1.40–1.73)	0.055
SBP, mmHg	0 (0)	112 (100–126)	115 (98–126)	112 (101–127)	0.916
DBP, mmHg	0 (0)	65 (57–76)	70 (62–75)	63 (57–76)	0.250
HR, 1/min	0 (0)	70 (62–80)	72 (67–86)	70 (61–79)	0.163
Hypertension	0 (0)	49 (38)	16 (53)	33 (33)	0.072
Diabetes	0 (0)	30 (23)	15 (50)	15 (15)	<0.001
Atrial fibrillation	0 (0)	21 (16)	5 (17)	16 (16)	1.000
History of CHD	0 (0)	29 (22)	13 (43)	16 (16)	0.004
History of non-cardiac PH	0 (0)	41 (32)	11 (37)	30 (30)	0.642
**Laboratory results**					
Hemoglobin, g/dL	0 (0)	13.4 (11.7–14.6)	14.1 (11.0–14.8)	13.3 (11.8–14.4)	0.205
BNP, pg/mL	37 (28)	168 (45–318)	315 (161–876)	113 (33–278)	0.001
NT-proBNP, pg/mL	94 (72)	326 (192–1,282)	1,971 (321–2,600)	293 (191–959)	0.175
**Echocardiographic parameters**					
IVSd, mm	0 (0)	10 (9–11)	11 (9–12)	10 (9–11)	0.213
LVPWd, mm	0 (0)	10 (9–11)	10 (8–12)	10 (9–11)	0.380
LVIDd, mm	0 (0)	47 (41–56)	56 (47–63)	46 (41–51)	<0.001
LVIDs, mm	0 (0)	31 (26–45)	48 (34–56)	29 (25–35)	<0.001
LVMi, g/m^2	3 (2)	102.5 (81.5–141.8)	141.0 (101.9–159.3)	96.5 (78.4–132.2)	<0.001
LVEDVi, mL/m^2	103 (79)	74.2 (56.2–97.5)	62.7 (39.3–92.7)	74.2 (57.1–97.5)	0.389
LVESVi, mL/m^2	103 (79)	32.3 (16.3–71.7)	23.8 (12.7–61.3)	32.3 (22.0–71.7)	0.418
LVEF, %	0 (0)	61.9 (37.3–69.9)	31.5 (28.0–49.3)	64.6 (47.9–71.1)	<0.001
LAVi, mL/m^2	22 (17)	35.5 (25.1–48.6)	39.4 (30.3–57.2)	33.4 (23.5–46.4)	0.068
LARS, %	6 (5)	17.2 (10.7–27.8)	9.8 (7.3–14.3)	21.4 (13.6–32.1)	<0.001
Moderate or greater AS	40 (31)	8 (9)	1 (4)	7 (11)	0.430
Moderate or greater AR	0 (0)	10 (8)	1 (3)	9 (9)	0.452
Moderate MR	0 (0)	21 (16)	8 (27)	13 (13)	0.133
E, cm/s	0 (0)	70 (51–90)	80 (60–104)	70 (51–85)	0.103
E-DT, ms	1 (1)	192 (150–238)	171 (146–217)	195 (159–246)	0.038
A, cm/s	22 (17)	70 (49–84)	57 (47–83)	70 (50–86)	0.448
E/A	23 (18)	0.9 (0.6–1.4)	0.9 (0.6–1.6)	0.9 (0.6–1.4)	0.665
IVRT, ms	75 (58)	89 (73–114)	89 (80–108)	90 (70–113)	0.880
e’ (septal), cm/s	0 (0)	5.5 (3.7–7.3)	4.3 (3.2–5.2)	6.0 (4.2–7.6)	<0.001
E/e’ (septal)	0 (0)	13.1 (9.7–16.8)	17.2 (14.0–24.1)	12.0 (9.0–14.8)	<0.001
e’ (lateral), cm/s	52 (40)	8.4 (6.7–11.4)	6.9 (5.9–8.1)	9.0 (7.2–11.7)	0.139
E/e’ (lateral)	52 (40)	8.3 (5.9–10.8)	10.2 (8.6–11.9)	7.9 (5.8–10.2)	0.180
e’ (average), cm/s	52 (40)	7.3 (5.6–9.2)	5.5 (5.0–6.2)	7.8 (6.4–9.6)	0.092
E/e’ (average)	52 (40)	9.2 (7.2–11.9)	11.1 (9.5–14.3)	8.9 (7.1–11.3)	0.068

Supplemental Table 14. Continued

	Missing n (%)	All patients n=130	Elevated LVFP n=30	Normal LVFP n=100	P-value
TRV, m/s	5 (4)	2.69 (2.24–3.20)	2.84 (2.42–3.10)	2.60 (2.20–3.20)	0.407
PASP, mmHg	63 (48)	37 (26–49)	44 (37–59)	37 (26–48)	0.259
2016 ASE/EACVI guidelines	0 (0)				
Normal diastolic function		9 (7)	0 (0)	9 (9)	
Indeterminate diastolic function		6 (5)	0 (0)	6 (6)	
DD with normal LAP		52 (40)	11 (37)	41 (41)	
DD with indeterminate LAP		33 (25)	9 (30)	24 (24)	
DD with elevated LAP		30 (23)	10 (33)	20 (20)	0.172
2025 ASE guidelines	0 (0)				
Normal diastolic function		9 (7)	1 (3)	8 (8)	
DD with normal LAP		59 (45)	6 (20)	53 (53)	
DD with indeterminate LAP		12 (9)	4 (13)	8 (8)	
DD with elevated LAP		50 (38)	19 (63)	31	0.003
DL-predicted probability of DD	0 (0)	0.98 (0.60–0.99)	0.99 (0.98–0.99)	0.93 (0.24–0.99)	<0.001
**Invasive hemodynamic assessment**					
PCWP, mmHg	0 (0)	10.0 (7.0–14.8)	20.0 (17.0–25.8)	8.0 (5.8–11.0)	<0.001

Continuous variables are presented as median (interquartile range) and categorical variables as n (%). Comparisons between subgroups with elevated and normal LVFP were performed using unpaired Student's t-test or Mann-Whitney U test for continuous variables, Chi-squared or Fisher's exact test for categorical variables, as appropriate.

Abbreviations as in Supplemental Tables 1, 2, and 3.

Supplemental Figure 1. Prognostic value of the guidelines and the DL model in multivariable Cox regression analysis for predicting the composite endpoint

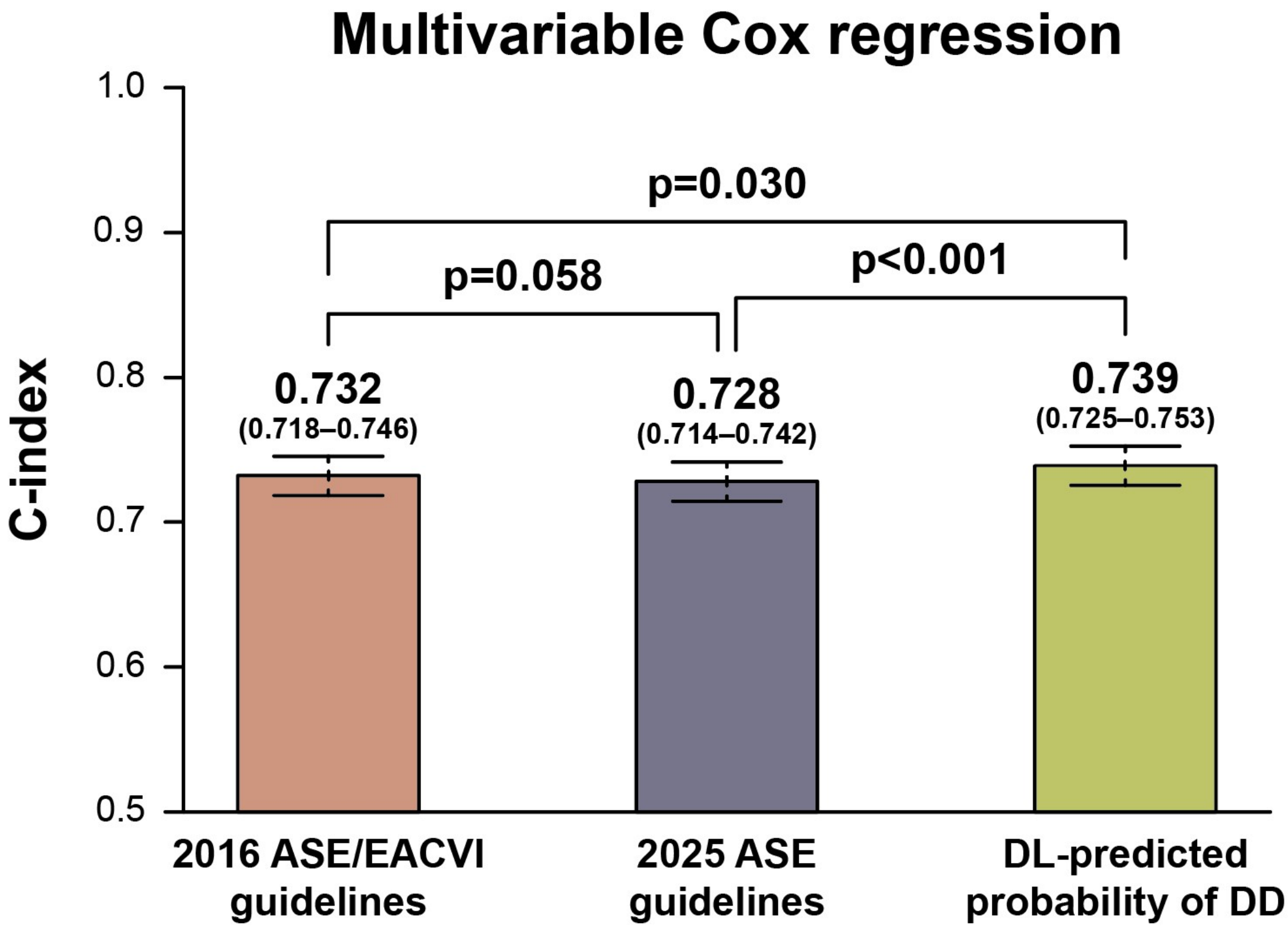

C-indices were calculated from multivariable Cox regression models that included age, sex, hypertension, diabetes, atrial fibrillation, chronic kidney disease, history of heart failure, history of coronary heart disease, history of non-cardiac pulmonary hypertension, and history of stroke, along with either the 2016 guideline-based classification, the 2025 guideline-based classification, or the DL-predicted probability of DD.

ASE – American Society of Echocardiography, EACVI – European Association of Cardiovascular Imaging, DD – diastolic dysfunction, DL – deep learning

Supplemental Figure 2. Time-dependent ROC curves of the guidelines and the DL model for predicting the composite endpoint of HF hospitalization or all-cause death

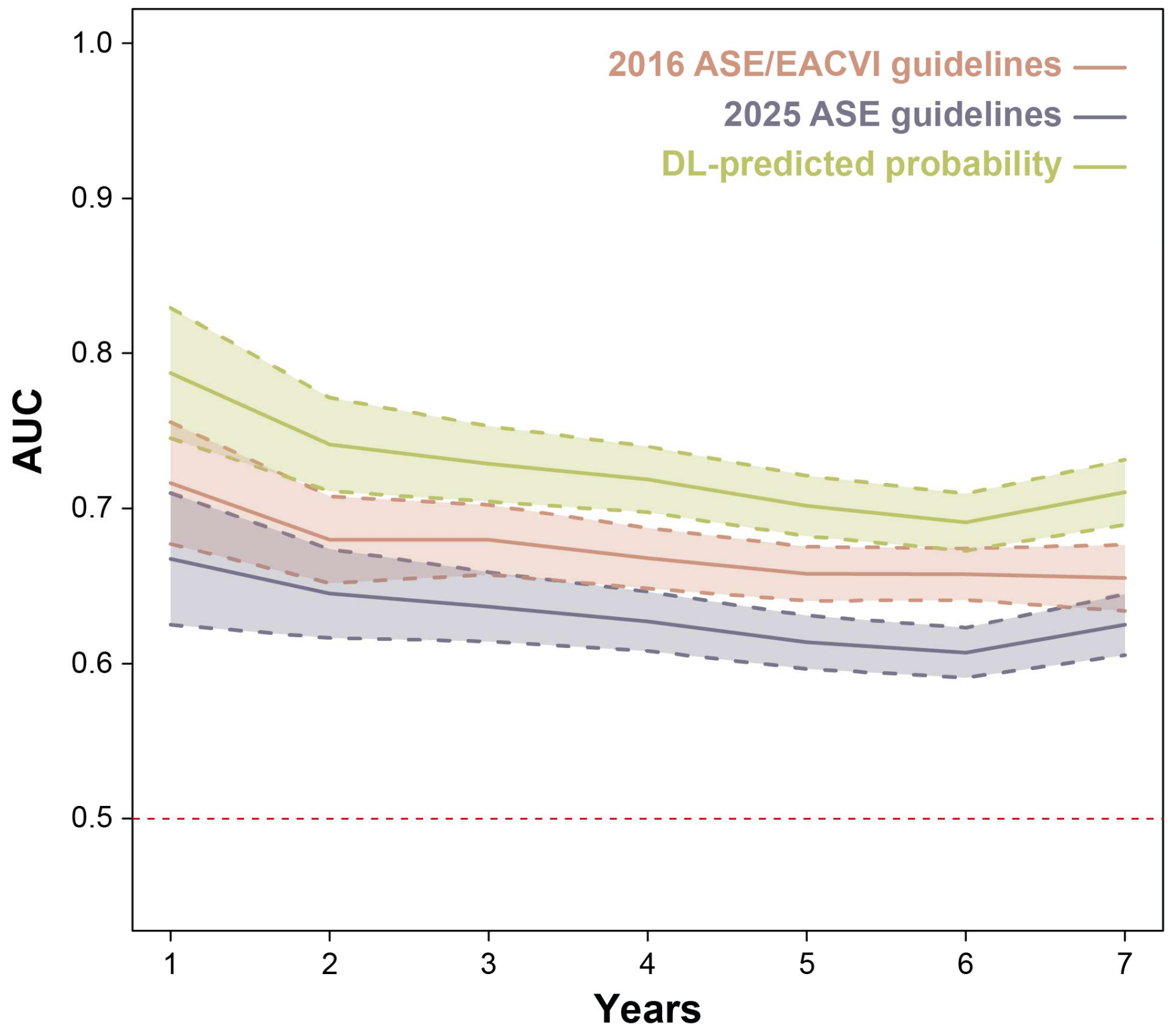

Cumulative time-dependent ROC curves were estimated using inverse probability of censoring weighting and are plotted with 95% confidence bands.

AUC – area under the receiver operating characteristic curve, ROC – receiver operating characteristic; other abbreviations as in Supplemental Figure 1.

Supplemental Figure 3. Prognostic value of the guidelines and the DL model in univariable analyses for predicting HF hospitalization and all-cause death separately

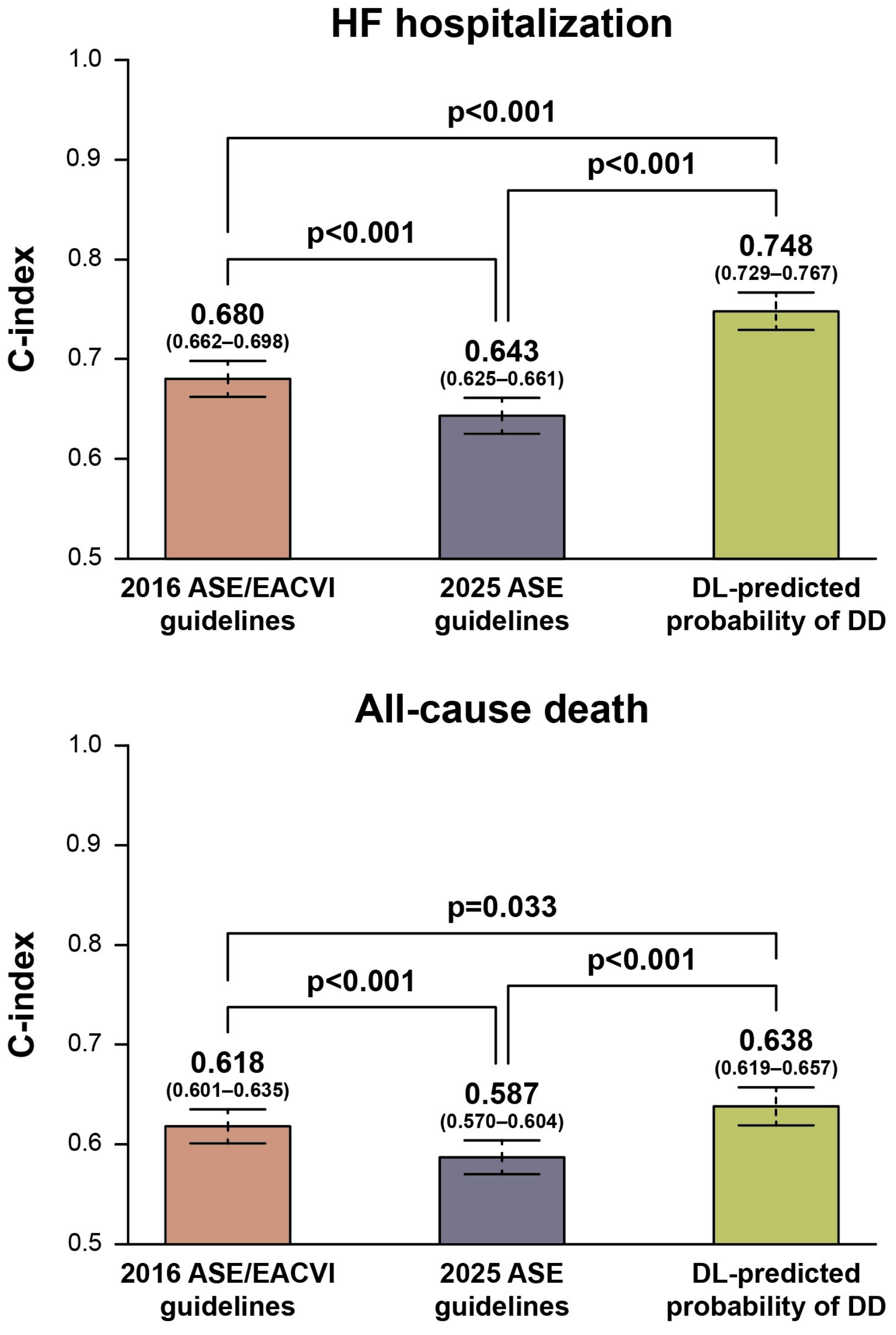

For HF hospitalization, C-indices were calculated from univariable Fine-Gray competing risk regression models (with all-cause death treated as the competing event) that included either the 2016 or 2025 guideline-based classification or the DL-predicted probability of DD as the sole predictor. For all-cause death, C-indices were calculated from univariable Cox regression models using the same predictor structure.

HF – heart failure; other abbreviations as in Supplemental Figure 1.

Supplemental Figure 4. ROC curves showing the performance of the guidelines and the DL model in detecting elevated LVFP after excluding patients with atrial fibrillation

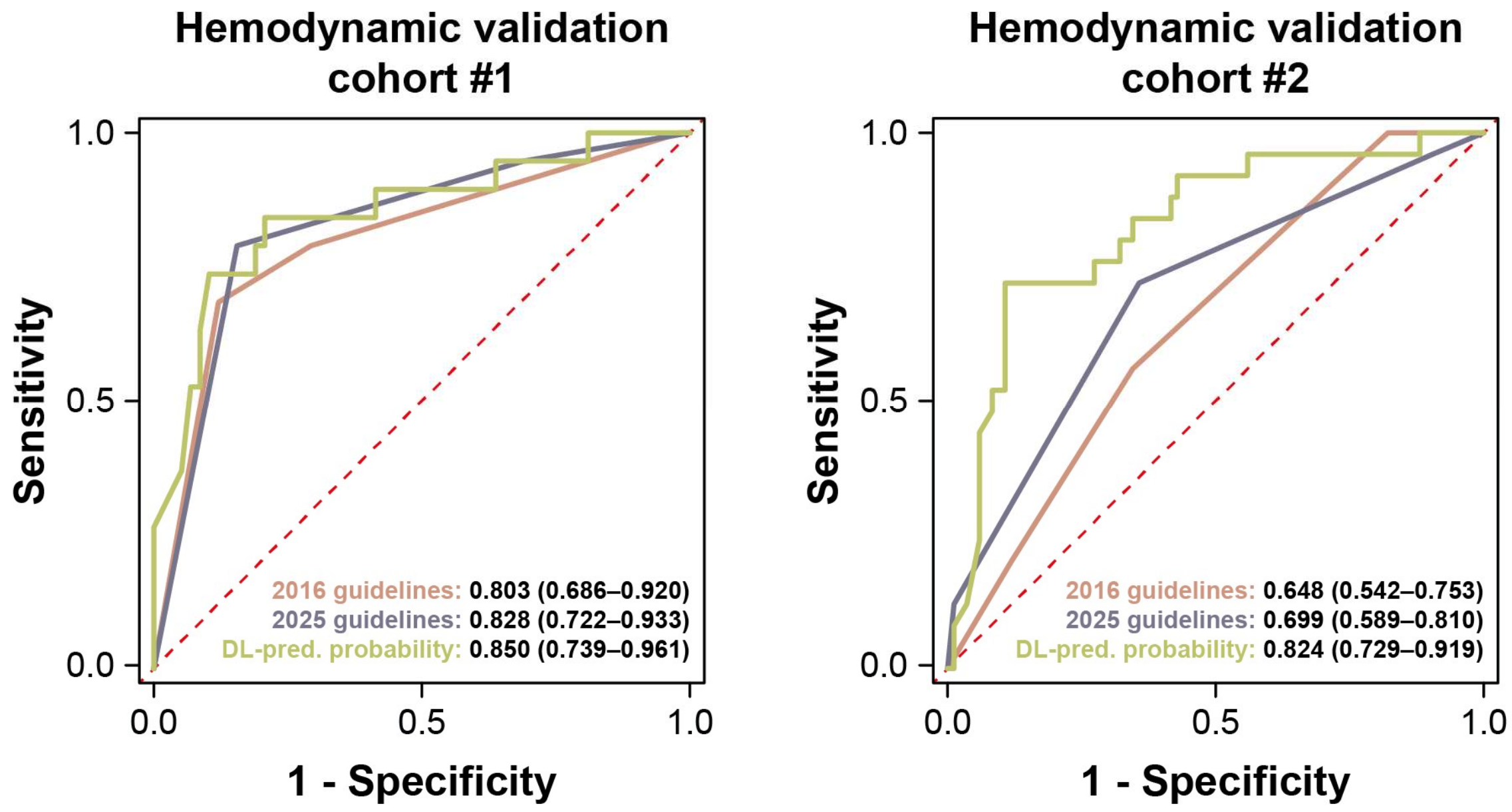

In this sensitivity analysis, 6 of 83 (7%) and 21 of 130 (16%) patients with atrial fibrillation were excluded from the first and second hemodynamic validation cohorts, respectively. AUCs are reported with 95% CIs.

LVFP – left ventricular filling pressure; other abbreviations as in Supplemental Figures 1 and 2.

Supplemental Figure 5. ROC curves showing the performance of the guidelines and the DL model in detecting elevated LVFP after excluding patients with both IVRT and LARS missing

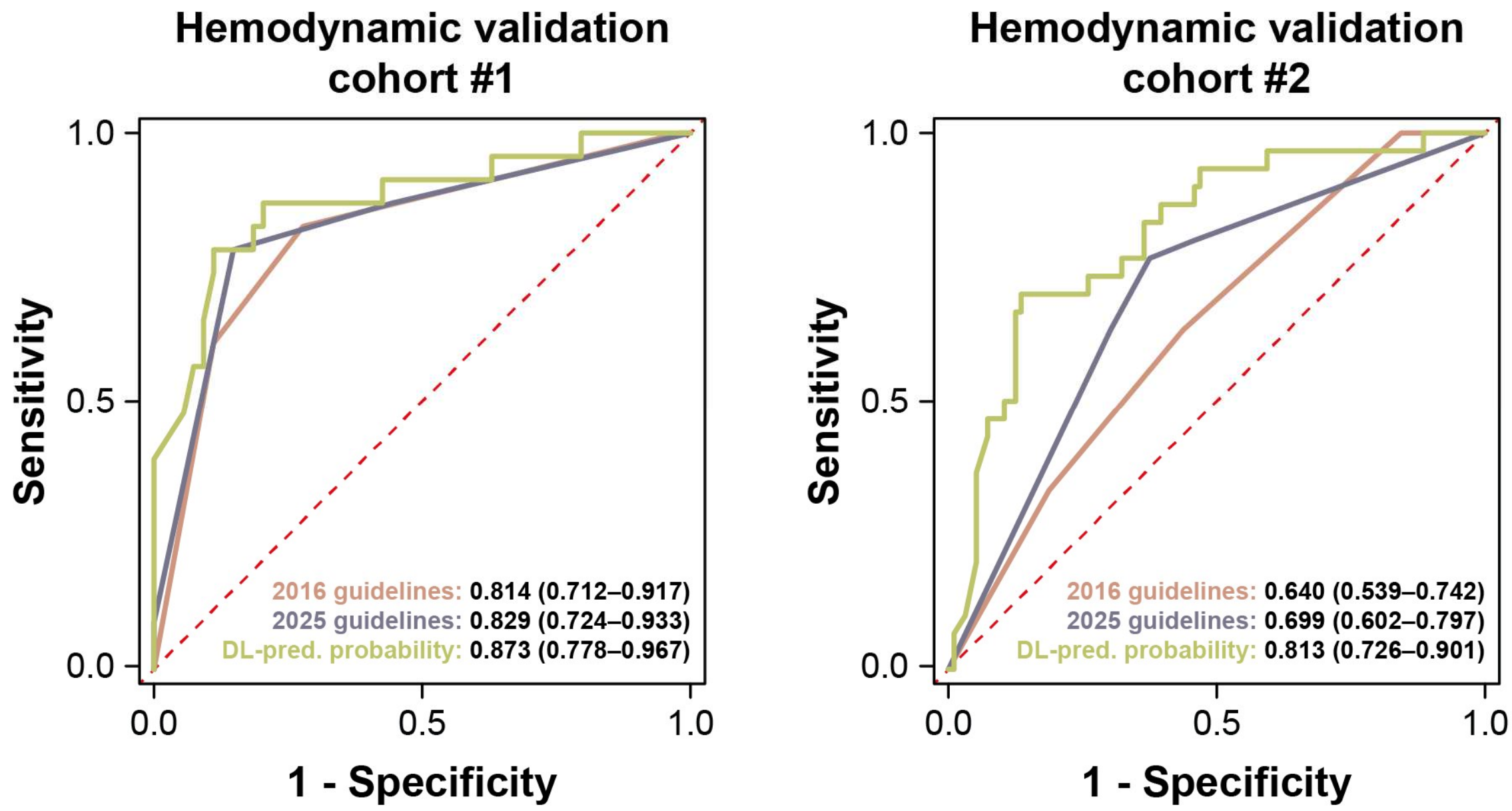

In this sensitivity analysis, 6 of 83 (7%) and 4 of 130 (3%) patients with both IVRT and LARS missing were excluded from the first and second hemodynamic validation cohorts, respectively. AUCs are reported with 95% CIs.

IVRT – isovolumic relaxation time, LARS – left atrial reservoir strain; other abbreviations as in Supplemental Figures 1, 2, and 4.
